# Comprehensive characterization and RNA-Seq profiling of the HD-Zip transcription factor family in soybean (*Glycine max*) during dehydration and salt stress

**DOI:** 10.1186/1471-2164-15-950

**Published:** 2014-11-03

**Authors:** Vikas Belamkar, Nathan T Weeks, Arvind K Bharti, Andrew D Farmer, Michelle A Graham, Steven B Cannon

**Affiliations:** Interdepartmental Genetics, Iowa State University, Ames, IA 50011 USA; Department of Agronomy, Iowa State University, Ames, IA 50011 USA; United States Department of Agriculture - Agricultural Research Service, Corn Insects and Crop Genetics Research Unit, Ames, IA 50011 USA; National Center for Genome Resources, Santa Fe, NM 87505 USA

**Keywords:** Soybean, HD-Zip, Transcription factor, Gene family, Whole-genome duplication, RNA-Seq, Dehydration stress, Salt stress, Abiotic stress

## Abstract

**Background:**

The homeodomain leucine zipper (HD-Zip) transcription factor family is one of the largest plant specific superfamilies, and includes genes with roles in modulation of plant growth and response to environmental stresses. Many HD-Zip genes are characterized in *Arabidopsis* (*Arabidopsis thaliana*), and members of the family are being investigated for abiotic stress responses in rice (*Oryza sativa*), maize (*Zea mays*), poplar (*Populus trichocarpa*) and cucumber (*Cucmis sativus*). Findings in these species suggest HD-Zip genes as high priority candidates for crop improvement.

**Results:**

In this study we have identified members of the HD-Zip gene family in soybean cv. ‘Williams 82’, and characterized their expression under dehydration and salt stress. Homology searches with BLASTP and Hidden Markov Model guided sequence alignments identified 101 HD-Zip genes in the soybean genome. Phylogeny reconstruction coupled with domain and gene structure analyses using soybean, *Arabidopsis*, rice, grape (*Vitis vinifera*), and *Medicago truncatula* homologues enabled placement of these sequences into four previously described subfamilies. Of the 101 HD-Zip genes identified in soybean, 88 exist as whole-genome duplication-derived gene pairs, indicating high retention of these genes following polyploidy in *Glycine* ~13 Mya. The HD-Zip genes exhibit ubiquitous expression patterns across 24 conditions that include 17 tissues of soybean. An RNA-Seq experiment performed to study differential gene expression at 0, 1, 6 and 12 hr soybean roots under dehydration and salt stress identified 20 differentially expressed (DE) genes. Several of these DE genes are orthologs of genes previously reported to play a role under abiotic stress, implying conservation of HD-Zip gene functions across species. Screening of HD-Zip promoters identified transcription factor binding sites that are overrepresented in the DE genes under both dehydration and salt stress, providing further support for the role of HD-Zip genes in abiotic stress responses.

**Conclusions:**

We provide a thorough description of soybean HD-Zip genes, and identify potential candidates with probable roles in dehydration and salt stress. Expression profiles generated for all soybean genes, under dehydration and salt stress, at four time points, will serve as an important resource for the soybean research community, and will aid in understanding plant responses to abiotic stress.

**Electronic supplementary material:**

The online version of this article (doi:10.1186/1471-2164-15-950) contains supplementary material, which is available to authorized users.

## Background

Plants sense and respond to environmental variations in temperature, nutrient availability, water level, and light conditions. The homeodomain leucine zipper (HD-Zip) transcription factors play a significant role in regulating plant growth adaptation responses by integrating developmental and environmental signals. Homeodomain leucine zipper (HD-Zip) transcription factors have been found exclusively in the plant kingdom [1, 2], the only exception being the recent identification in the charophycean algae [3]. The characteristic feature of the HD-Zip gene family is the association of homeodomain (HD) and the leucine zipper (LZ) motif in a single protein. In other kingdoms, they are present as domains of distinct proteins. The homeodomain is a ~60 amino acid DNA binding domain composed of a helix-turn-helix structure that folds into three characteristic alpha-helices, capable of interacting specifically with DNA [2]. The LZ motif is a dimerization motif and is located immediately after the HD. The LZ motif allows the formation of homo- and hetero-dimers that are required for binding to DNA. The HD-Zip transcription factors can be subdivided into four subfamilies: HD-Zip I to IV, based on distinct sequence features (DNA-binding domains and additional conserved motifs that are specific to each of the subfamilies), and distinct functions of proteins from each of the subfamilies (for reviews, see [1, 4]).

The HD-Zip superfamily has been analyzed in several species including *Arabidopsis* (*Arabidopsis thaliana*) [1, 5, 6], rice (*Oryza sativa*) [4, 7], maize (*Zea mays*) [8], poplar (*Populus trichocarpa*) [9], and the HD-Zip I and IV genes in cucumber (*Cucumis sativus*) [10, 11]. However, functional characterization studies have been limited to the model plant *Arabidopsis*, while a few selected genes have been investigated in other species [1, 4]. A subset of the HD-Zip genes have recently been described in soybean (*Glycine max*) [12]. HD-Zip genes are involved in several abiotic stress responses, meristem regulation, photomorphogenesis, and root development [1, 4]. The HD-Zip I genes have been investigated for their roles in water deficit and salt stress responses. The HD-Zip I *Arabidopsis* genes *ATHB7* and *ATHB12*, and their orthologs in other species, including *HaHB4* from sunflower (*Helianthus annuus*), *NaHD20* in *Nicotiana attenuatta*, and *OsHOX6* in rice (*Oryza sativa*), have increased expression under water-stress conditions [7, 13–15]. ATHB7 and ATHB12 act as negative regulators of growth and development by reducing plant growth under water-deficit conditions [13, 16, 17]. *HaHB4* delays the onset of senescence when expressed in *Arabidopsis* [18, 19]. The *Arabidopsis* HD-Zip I genes *ATHB5* and *ATHB6*, and the homologs in *Craterostigma plantagineum CpHB5*, *6*, *7* and *CpHB8*, are involved in water deficit stress [20]. ATHB5 acts as a positive regulator of ABA responsiveness at the seedling stage, with elevated levels of *ATHB5* resulting in higher ABA responsiveness. On the contrary, ABA reduces the wild-type expression of *ATHB5*, indicating *ATHB5* is part of a negative feedback loop regulating ABA sensitivity in the germinating seedlings [21]. This implies ATHB5 mediates an initial response of the seedling to an ABA signal imposed (for instance, seedling development under limited-term water-deficit conditions) - but reduces the response to extended water stress. The *Arabidopsis* gene *ATHB6* has increased expression under water deficit stress [22]. *Arabidopsis* plants overexpressing ATHB6 display lowered stomatal closure and reduced inhibition of germination by ABA [23] - the characteristics of the ABA-insensitive mutant *abi1* and *abi2* [24]. Deng et al. [25] suggested that ATHB6 acts as a negative regulator of ABA response under water deficit stress.

A recent study in maize found all 17 HD-Zip I genes differentially expressed (DE) under drought stress [8]. The *Arabidopsis* genes *ATHB21*, *ATHB40* and *ATHB53* and the *M. truncatula* gene *MTHB1* are induced by salt stress [4]. The over-expression of MTHB1 reduces lateral root emergence. Ariel et al. [26] proposed reduced lateral root growth as a mechanism to reduce the exposure of the roots to high saline soil. The *Arabidopsis* gene *HAT22*, the rice genes *OsHOX11* and *OsHOX27*, and the *C. plantagineum* genes *CpHB1* and *CpHB2*, all in HD-Zip II, are induced by water stress [7, 20, 27]. Thus, it is evident that members of the HD-Zip I and II are enriched for genes that are involved in water deficit and salt stress. The emphasis in the literature has focused on HD-Zip I proteins for their role in abiotic stress, while systematic characterization of genes from the other subfamilies has been lacking. A recent study in rice shows the importance of investigating genes from other subfamilies. Yu et al. [28] demonstrated the overexpression of a HD-Zip IV gene (HDG11) confers drought tolerance, and increases yield under both normal and drought conditions. With the utilization of high throughput sequencing techniques such as RNA-Seq, it is possible to investigate the expression of HD-Zip genes belonging to all subfamilies in the same experiment, and identify potential candidates for functional characterization studies.

The identification and classification of HD-Zip genes in prior studies has been based on homology searches, well-conserved domains and motifs in each of the subfamilies, and on conserved gene structures among subfamily members [5–11]. The availability of whole genome sequence information for increasing numbers of angiosperm species has enabled utilization of evolutionary relationships among the species to help characterize HD-Zip genes. Evolutionary relationships among species in a gene family analysis can be combined with whole genome duplication (WGD) histories. The eudicots *Arabidopsis*, grape, soybean and *M. truncatula* share a common “gamma” genome triplication event that occurred around 117 million years ago (Mya), early in the eudicot evolution [29, 30]. The *Arabidopsis* lineage shows a signal for two additional rounds of WGD events within the last 70 million years [30, 31]. Soybean and *Medicago* share a common legume-specific WGD event approximately 59 Mya [32, 33], and soybean has undergone an additional glycine-specific genome duplication event around 13 Mya [30, 32]. Rice shows evidence of two rounds of WGD events [30]. The grape genome has undergone a genome triplication event, but lacks a recent WGD event [34]. Conceptually, a single-copy gene in the ancestor of angiosperm plants and retained after every WGD event would give rise to the following numbers of homologous genes: 3 in grape, 6 in *Medicago,* 4 in rice, and 12 each in soybean and *Arabidopsis*. There is also evidence for two even older WGDs: one at around 320 Mya, prior to the separation of angiosperms and gymnosperms and referred to as the “ancestral seed plant WGD”; and another at around 190 Mya, predating the origin of angiosperms and termed the “ancestral angiosperm WGD” [31]. Per this model of WGDs, an angiosperm gene family is typically comprised of four old angiosperm clades, assuming a starting point of one gene copy in the ancestor of seed plants. We examine the HD-Zip family in the context of this hypothesized history of WGDs, and provide insights into evolutionary history of each of the subfamilies relative to these WGD events.

In this study, we have 1) identified all putative HD-Zip genes in the soybean genome and placed them into their respective subfamilies; 2) provided phylogenetic relationships among HD-Zip proteins from eight species that include six eudicots: poplar, cucumber, *Arabidopsis*, grape, soybean and *M. truncatula*, and two moncots: rice and maize; 3) characterized the structures of all HD-Zip genes; 4) described the genomic organization, tracing the expansion of the gene family through WGD events; 5) presented gene expression data for all HD-Zip genes in 24 conditions including at least 17 different tissues of soybean; 6) provided RNA-Seq based gene expression profiles of all soybean genes including HD-Zip genes, in the roots under normal conditions and dehydration and salt stress after 0, 1, 6 and 12 hr treatments; and 7) identified genes that may participate in HD-Zip gene pathways by screening HD-Zip promoters for conserved motif of transcription factor binding sites (TFBSs).

## Methods

### Homology searches, multiple sequence alignments, and phylogenetic analysis

The sequences of 47 HD-Zip (17 HD-Zip I, 9 HD-Zip II, 5 HD-Zip III and 16 HD-Zip IV) proteins of *A. thaliana* (TAIR8_genome_release, 11/30/09) described in Ariel et al. [1], were obtained from TAIR [35]. The proteomes of four other sequenced angiosperms *G. max* (assembly v1.01, JGI Glyma1.0 annotation), *M. truncatula* (v 3.5.1), *O. sativa* (MSU Release 6.0) and *V. vinifera* (12X March 2010 release) were obtained from the respective repositories for these genomes and BLAST databases were built for each of them on our local server. A BLASTP v2.2.22 (protein-protein BLAST) [36] search with a threshold of 1E-10 was used for initial identification of the homologous *Arabidopsis* HD-Zip genes in each of the genomes described above. The multiple sequence alignment of the homologous sequences from the five species was performed using MUSCLE v3.8.31 [37]. The alignment was manually inspected and trimmed using SeaView v4.2.5 [38, 39] and BBEdit v8.7.2 respectively. A preliminary phylogenetic tree (not shown) encompassing four HD-Zip subfamilies was built using CLUSTAL v2.0.12 [40] and the tree was visually examined using FigTree v1.3.1 [41].

The probable HD-Zip genes belonging to each of the four subfamilies were identified based on the clustering of sequences with known HD-Zip genes from *Arabidopsis* in the preliminary phylogenetic tree. The outlier sequences that did not cluster with any *Arabidopsis* genes were temporarily excluded. The probable HD-Zip genes were then aligned using MUSCLE to build a profile Hidden Markov Model (HMM) separately for each subfamily using the hmmbuild program, implemented in the package HMMER v3.0b2 [42]. The probable HD-Zip sequences were re-aligned to the profile HMM using hmmalign, available in the tool HMMER, and were viewed in SeaView. Sequence logos were generated for each subfamily using the web tool WebLogo [43] to identify conserved regions in the alignments (Additional file 1: Figure S1, Additional file 2: Figure S2, Additional file 3: Figure S3 and Additional file 4: Figure S4). The HMM alignments were trimmed to retain the conserved regions (HMM “match states”) using BBEdit. The trimmed alignments were used to build the phylogenetic trees for each subfamily using the maximum likelihood method implemented in PhyML v3.0 [44] available at iPlant Collaborative [45] using default settings. The approximate likelihood ratio test (aLRT) branch support values [46] are displayed on the branches in percentages. The phylogenetic tree for each subfamily was displayed using FigTree. The rooting was inferred from Ariel et al. [1], angiosperm clade composition, and outgroup sequences from other subfamilies (data not shown).

The outlier sequences excluded based on the preliminary phylogenetic tree were used in a search against HMM of each subfamily using the hmmpfam available in the tool HMMER v2.3.2 and the membership of sequences in each subfamily was investigated. The process of generating a phylogenetic tree followed by excluding outlier sequences, re-alignments, building HMMs, re-aligning using HMM, and rebuilding the trees, was iterated several times for each subfamily. A phylogenetic tree with appropriate tree topology based on evolutionary relationship among the five species was then generated for each subfamily.

Lastly, we added HD-Zip I to IV sequences from maize and poplar, and HD-Zip I and IV sequences from cucumber that have recently been reported [8–11] to the final phylogenetic trees. This will allow the investigation of orthologous sequences from eight species that includes HD-Zip genes identified in all angiosperm species to date.

### Validation, structural characterization, and duplication history of HD-Zip genes

The HD-Zip subfamilies have remarkably well-conserved domains, motifs, and gene structures [1, 2, 4] that can be utilized to validate genes identified using phylogenetic analysis. All sequences identified as HD-Zip genes as well as outlier sequences (excluded after preliminary phylogenetic tree construction) were used as queries in a batch search [47] against Pfam 27.0, with an E-value threshold of 1E-3 to identify the conserved domains. The conserved motifs were investigated by examining the sequence logos that were generated using HMM sequence alignments of each subfamily. The gene structure was studied using the exon-intron organization in the pre-mRNA. The gene structures were rendered using the *G. max* cv. Williams 82 gene models (assembly v1.01, JGI Glyma1.0 annotation) that were downloaded from Phytozome [48] and using a modified version of the Bio-Graphics 2.25 feature_draw.pl script [49]. The genomic locations were obtained from the GFF file of *G. max* assembly v1.01, JGI Glyma1.0 annotation, and were displayed using chromosome visualization tool (CViT) [50]. The homoeologous HD-Zip gene pairs that are a result of the early-legume WGD event (~59 Mya), and the *Glycine*-specific duplication event (~13 Mya), were inferred from the phylogeny, as well as from the syntenic paralog pair information available for all soybean genes from the Joint Genome Institute (JGI) at Phytozome [51]. Paralogous genes resulting from tandem duplication events were identified based on their proximity on the same chromosome [52] and pairing in the same clade in the phylogenetic tree.

### Expression profiles of HD-Zip genes in 24 conditions (17 tissues) of soybean

An RNA-Seq atlas of *G. max* describing expression of genes in 24 conditions including at least 17 different tissues of soybean was reported by Severin et al. [53] and Libault et al. [54, 55]. The Reads/Kb/Million (RPKM) normalized data for 14 tissues investigated by Severin et al. are available for download and interactive analysis at SoyBase [56], and expression data for three additional tissues, and tissues infected with the bacterium *Bradyrhizobium japonicum* are available at SoyKB [57]. A gene was considered expressed if the RPKM value was greater than or equal to two in an expression atlas (modified criteria from [58]). The RPKM normalized read count data of expressed genes was log_2_-transformed and displayed in the form of heatmaps for each subfamily. The heatmap was generated in R [59] using the heatmap.2 function available in the gplots CRAN library. Genes in the heatmaps were ordered for consistency with the phylogeny.

### Plant material and stress experiment

The seeds of *G. max* cv. Williams 82 were germinated on moist germination paper and were allowed to grow until the v1 stage (first trifoliolate stage) in a growth chamber maintained at 77 F and 60% humidity throughout the experiment. The temperature and humidity were continuously monitored and maintained in the growth chamber. The salt treatment was applied by transferring the seedlings into 100 mM NaCl solution. For the dehydration treatment, plants were removed from the germination paper and left in air under water-limiting conditions to impose dehydration stress. Root tissue was harvested after 0, 1, 6 and 12 hr of stress treatments. Five plants per time point were maintained for each of the stress treatments. In order to verify the gradual imposition of salt stress treatment, electrical conductivity was measured in two fragments of germination paper, after harvesting root tissue from plants exposed to salt stress at each of the time points (data not shown). Total RNA was isolated using Qiagen RNeasy® Plant mini kit from three biological replicates per time point per the manufacturer’s protocol. The RNA samples were treated with Ambion® TURBO DNA-free™ DNase to get rid of any DNA contamination in the RNA samples. The RNA samples were inspected for their quality and quantity using NanoDrop® spectrophotometer and Qubit® fluorometer.

### Sequencing, data processing, gene expression analysis and annotation under stress conditions

Total RNA from 21 samples that includes three control samples (0 hr), and three biological replicates for each of the three time points 1, 6 and 12 hr under dehydration and salt stress was sent to the National Center for Genome Resources (Santa Fe, NM, USA) for sequencing on Illumina® HiSeq 2000. Seven randomly chosen samples were multiplexed in each lane and three lanes of HiSeq 2000 were utilized to generate single-end short-reads of 1×50 bp lengths. The reads were aligned with GSNAP [60] using default settings with a maximum of 4 mis-matches allowed against the *Glycine max* genome assembly and annotation v1.01 from Phytozome (JGI Glyma1.0 gene calls). The uniquely mapped reads that mapped to a single location in the genome were analyzed for differential gene expression between the control and treatment samples using the R package DESeq v1.7.10 [61]. A gene was considered to be DE if it satisfied the following three stringent filtering criteria: (1) *P*-value adjusted for multiple testing correction using Benjamini and Hochberg method [62] to be less than 0.05, (2) two fold or greater fold change, (3) residual variance quotients of both the control and treatment samples of less than 20. The residual variance criterion was used to filter genes that have significant variation between replicates, per recommendations in the DESeq manual (Released April 20, 2011). The raw and the normalized read counts, and the sequence data has been deposited in NCBI’s Gene Expression Omnibus [63, 64] and are accessible through the GEO series accession number GSE57252.

The DE genes were annotated using the top *Arabidopsis* hit, and the corresponding gene ontology (GO) biological process and molecular function terms were inferred [65]. The DE genes under dehydration and salt stress were then screened separately for overrepresented GO terms against all soybean genes using Fisher’s exact test [66] and Bonferroni [67] corrected significance value of less than 0.05. The overrepresented GO terms were enriched at the second level using BLAST2GO v.2.7.1 [68] and a reduced representation of enriched GO terms was obtained. The DE genes were also annotated using the SoyDB [69, 70] transcription factor (TF) database, and Fishers’s exact test followed by Bonferroni correction was utilized to determine the overrepresented TF classes under each of the stress conditions.

### Screening of HD-Zip gene promoters for conserved motifs of transcription factor binding sites (TFBSs)

For the purpose of this study, the one kilobase (kb) region upstream of the annotated transcription start site for each gene was evaluated for promoter motifs. Promoter sequences were retrieved using custom Perl scripts for all gene models in the soybean genome. Promoter sequences that were either less than one kb or included two or more Ns were excluded from the analysis. The program Clover [71] was used to scan through a database of known motifs in TRANSFAC® v. 2010.4 [72]. Promoters of HD-Zip genes belonging to each subfamily were scanned separately for enriched motifs against a background of all soybean gene promoters, with a *P*-value threshold of 0.05 and an individual motif hit score of greater than or equal to 6. Similarly, promoters of genes that were DE in at least one of the three time points under dehydration and salt stress were screened to identify overrepresented motifs under each of the stress treatments. The overrepresented motifs were filtered to include only plant motifs. A comparison was made between motifs that were overrepresented in the promoters of HD-Zip genes belonging to each of the subfamilies and dehydration and salt stress treatments.

## Results

### Classification of HD-Zip genes using phylogenetic analysis

**Figure 1:**
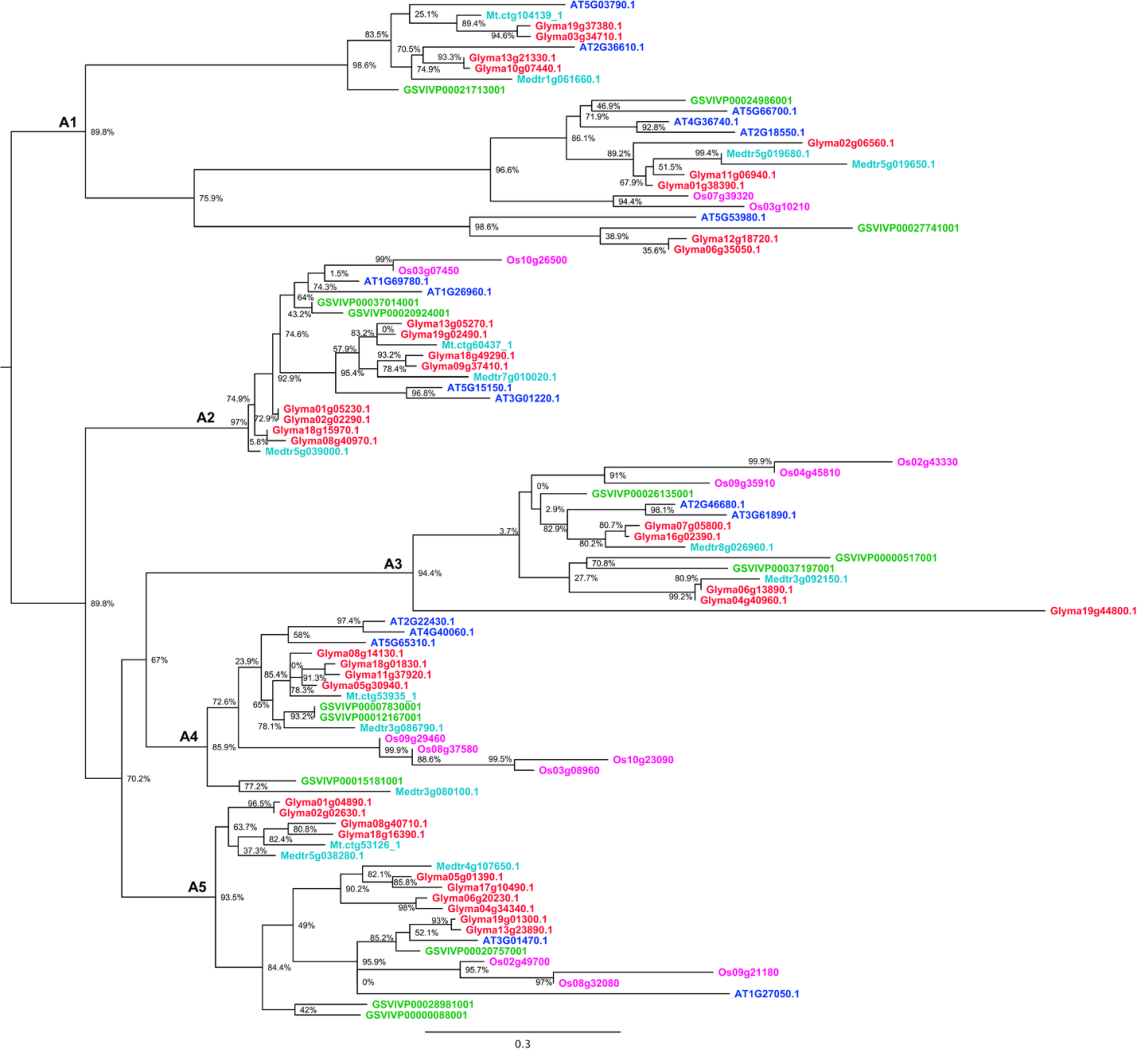
**Phylogenetic relationships of HD-Zip I proteins from soybean,** ***Medicago*** **,** ***Arabidopsis*** **, grape and rice.** The phylogenetic tree was built using the maximum likelihood method implemented in PhyML. The letters A1- A5 represent ancient angiosperm clades, based on whole genome duplication events, and the copy number of genes from each of the species. The branch support values estimated using approximate likelihood ratio test (aLRT) are displayed in percentages. Rooting of the tree was inferred from Ariel et al. [1], angiosperm clade composition, and outgroup sequences from other subfamilies. Genes from each of the species are highlighted in different colors, soybean (red), *Medicago* (light blue), *Arabidopsis* (dark blue), grape (green), and rice (Pink).

**Figure 2:**
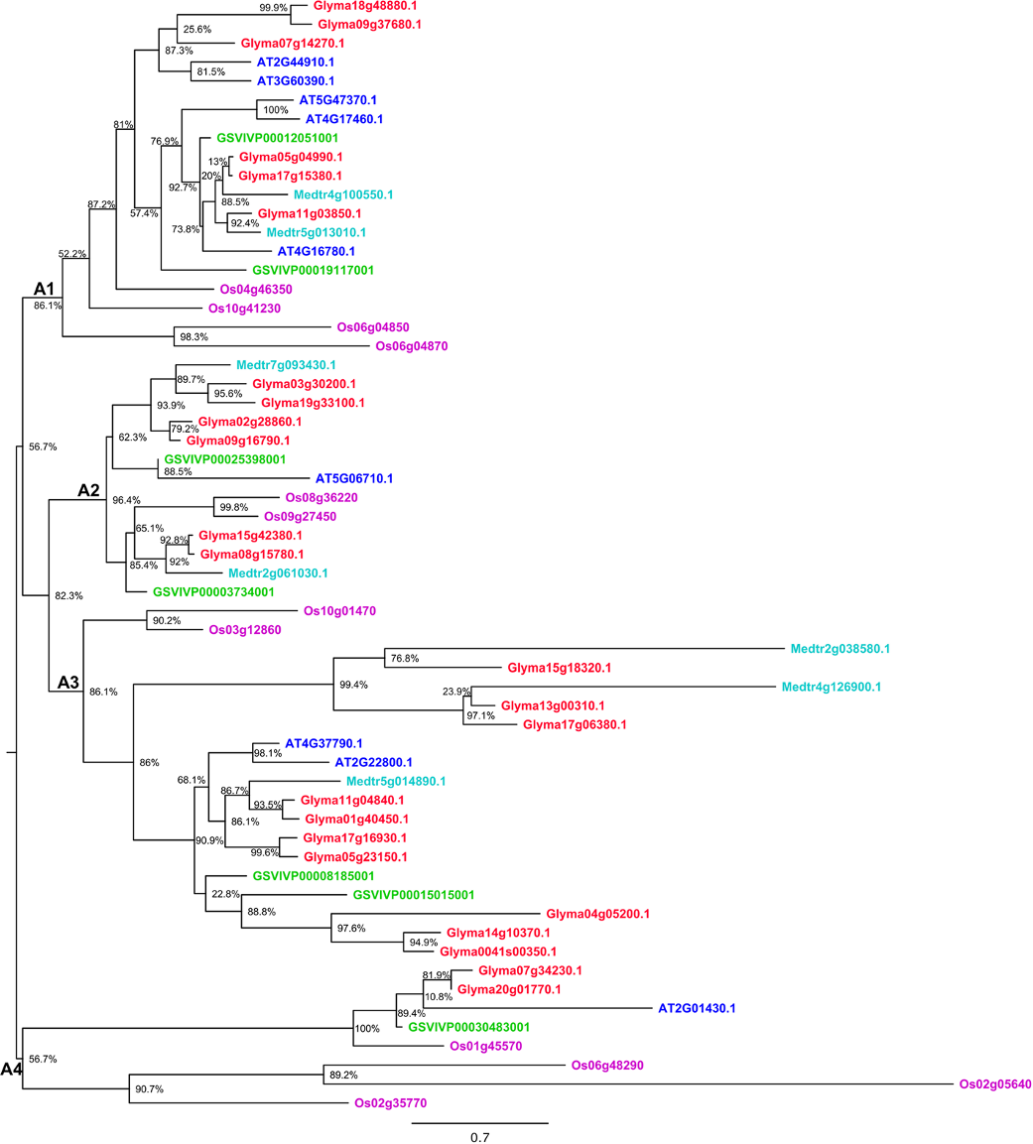
**Phylogenetic relationships of HD-Zip II proteins from soybean,** ***Medicago*** **,** ***Arabidopsis*** **, grape and rice.** The phylogenetic tree was built using the maximum likelihood method implemented in PhyML. The letters A1- A4 represent ancient angiosperm clades, based on whole genome duplication events, and the copy number of genes from each of the species. The branch support values estimated using approximate likelihood ratio test (aLRT) are displayed in percentages. Rooting of the tree was inferred from Ariel et al. [1], angiosperm clade composition, and outgroup sequences from other subfamilies. Genes from each of the species are highlighted in different colors, soybean (red), *Medicago* (light blue), *Arabidopsis* (dark blue), grape (green), and rice (Pink).

**Figure 3:**
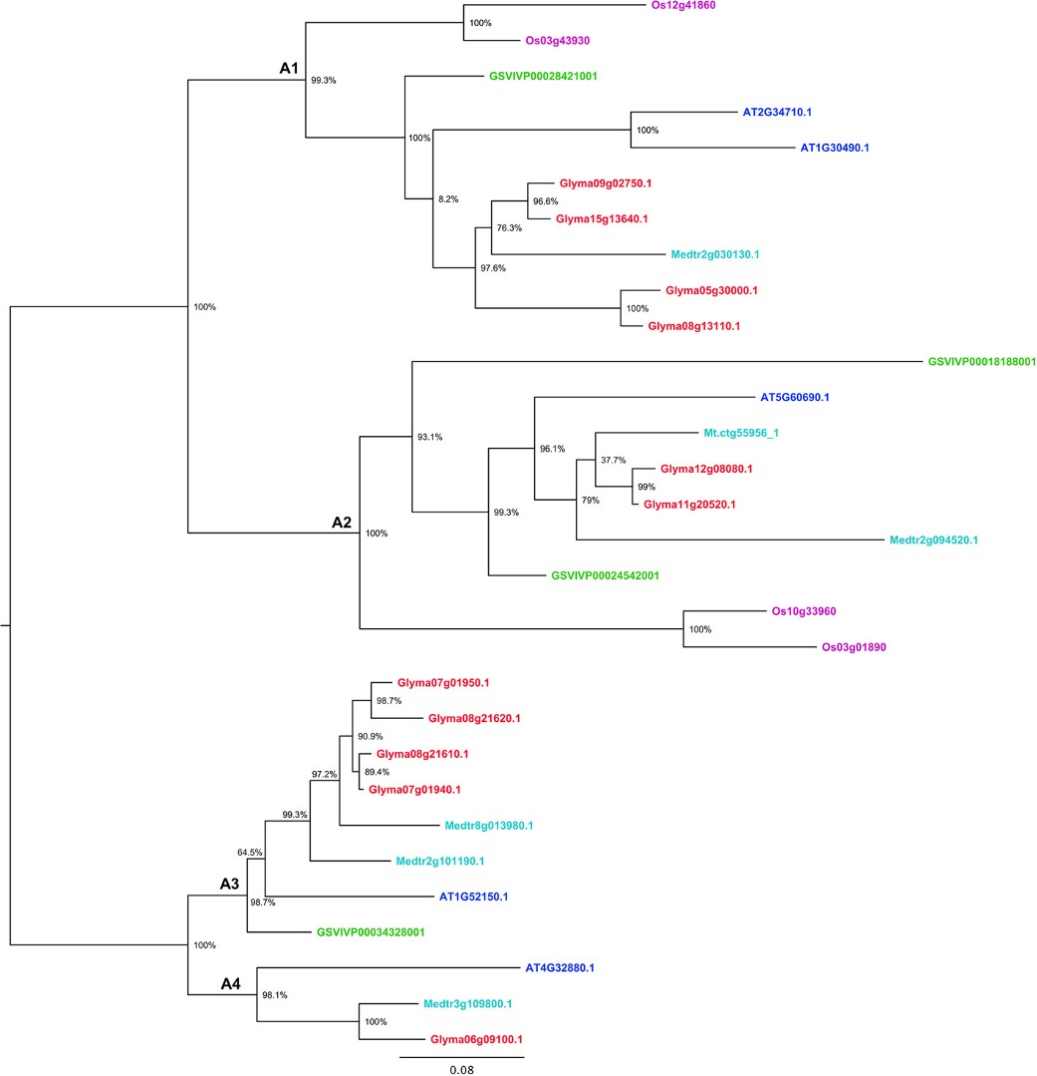
**Phylogenetic relationships of HD-Zip III proteins from soybean,** ***Medicago*** **,** ***Arabidopsis*** **, grape and rice.** The phylogenetic tree was built using the maximum likelihood method implemented in PhyML. The letters A1- A4 represent ancient angiosperm clades, based on whole genome duplication events, and the copy number of genes from each of the species. The branch support values estimated using approximate likelihood ratio test (aLRT) are displayed in percentages. Rooting of the tree was inferred from Ariel et al. [1], angiosperm clade composition, and outgroup sequences from other subfamilies. Genes from each of the species are highlighted in different colors, soybean (red), *Medicago* (light blue), *Arabidopsis* (dark blue), grape (green), and rice (Pink).

**Figure 4:**
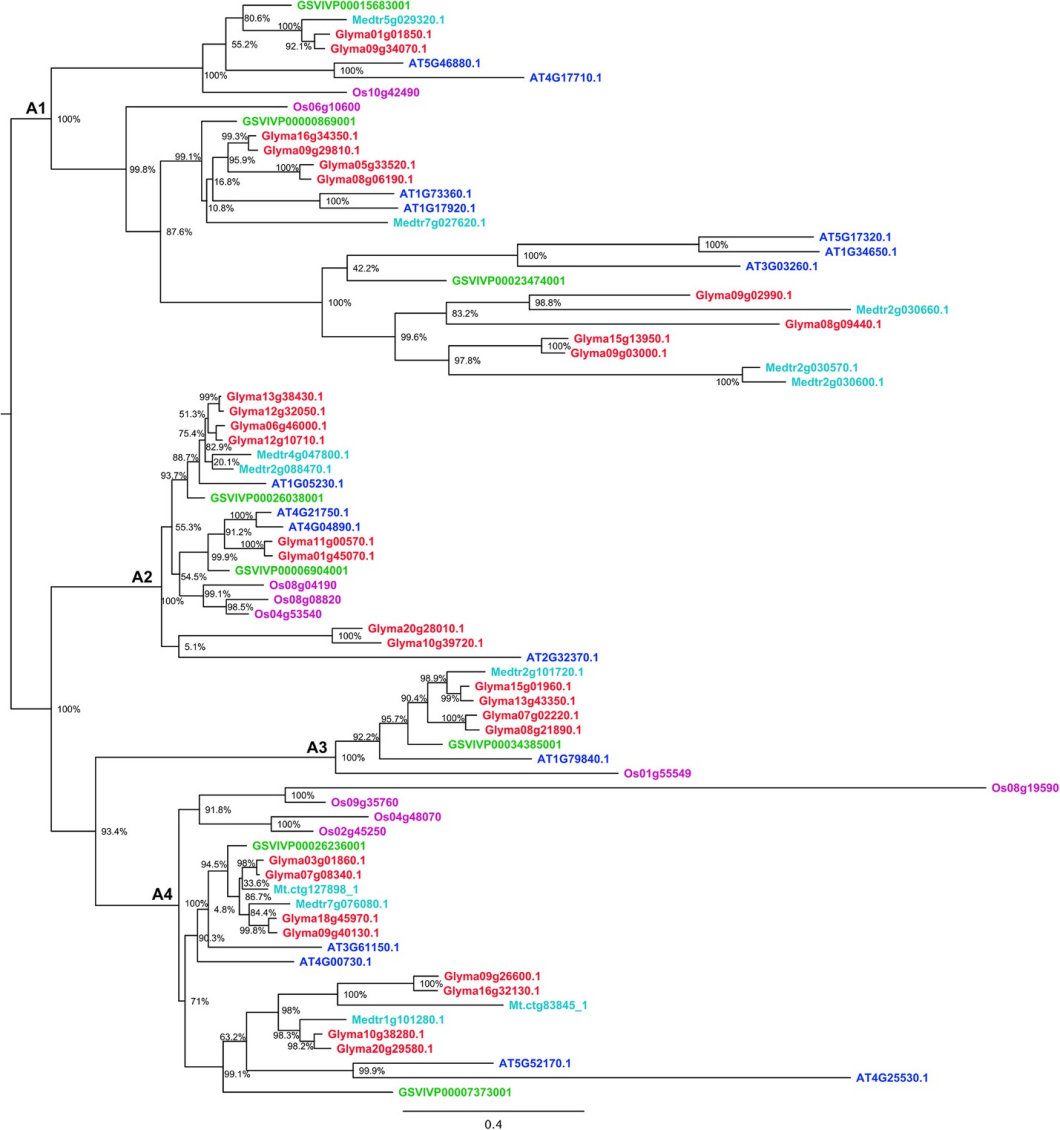
**Phylogenetic relationships of HD-Zip IV proteins from soybean,** ***Medicago*** **,** ***Arabidopsis*** **, grape and rice.** The phylogenetic tree was built using the maximum likelihood method implemented in PhyML. The letters A1- A4 represent ancient angiosperm clades, based on whole genome duplication events, and the copy number of genes from each of the species. The branch support values estimated using approximate likelihood ratio test (aLRT) are displayed in percentages. Rooting of the tree was inferred from Ariel et al. [1], angiosperm clade composition, and outgroup sequences from other subfamilies. Genes from each of the species are highlighted in different colors, soybean (red), *Medicago* (light blue), *Arabidopsis* (dark blue), grape (green), and rice (Pink). Genes Medtr5g005600.1 and Os01g57890 belong to the angiosperm clade “A2”. These two genes are not shown in the phylogeny because adding them significantly affects the topology.

A BLASTP search with the *Arabidopsis* HD-Zip genes against soybean, *M. truncatula*, grape and rice, followed by reconstruction of the phylogeny, clustered the sequences into four previously defined HD-Zip subfamilies (I to IV). HMMs for each subfamily were used to determine subfamily membership and refine alignments. The outlier sequences excluded from the preliminary tree (see methods for details) included six sequences that belonged to the HD-Zip IV subfamily and these were included in the final phylogenetic trees of the four subfamilies (Figures 1, 2, 3 and 4).

Based on the species clustering patterns and the number of copies of genes belonging to each species, we identified four old angiosperm clades in HD-Zip II, III and IV, and five clades in HD-Zip I (Figures 1, 2, 3 and 4). The topology of most of the angiosperm clades is generally consistent with the species tree. Typically the two legume species (soybean and *M. truncatula*) form a clade, with *Arabidopsis*, grape, and rice each as increasingly distant outgroups from the legume sequences in the clade. The number of copies of genes of each species in each angiosperm clade reflects the number of WGD events the species has undergone. For instance, four of the five angiosperm clades in HD-Zip I phylogeny included exactly three grape sequences – likely the result of the “gamma” triplication event that occurred around 117 Mya [29, 30], and the angiosperm clade A1 in the HD-Zip I phylogeny contains nine of the 12 possible soybean sequences - possibly the result of “gamma” triplication event (~117 Mya), and the legume- (~59 Mya) and *Glycine*-specific (~13 Mya) WGD events [30, 32]. We identified 101 genes in soybean, 47 in *Arabidopsis*, 33 in grape, and 41 each in *M. truncatula* and rice (Table 1). The highest gene retention rate (52.7%) among the five species is in HD-Zip IV, whereas the HD-Zip III has the lowest (20.3%) retention rate (Table 1). Although soybean has the highest number of genes, grape and rice have relatively higher retention rate of 64.7% and 60.3% respectively (Table 1). Arabidopsis has the lowest retention rate of 23.0%, whereas soybean and *M. truncatula* have intermediate retention rates of 49.5% and 40.2% respectively (Table 1). The varying rate of retention across the five species reflects the changes in the genomes of each of the species after WGD events. Overall the phylogenetic analysis together with the WGD histories helps clarify our understanding of the evolution of each of the subfamilies.

**Table 1:** **Number of HD-Zip genes observed (O), expected (E) and retained (R) among five angiosperm species**

	HD-Zip I (5)^a^			HD-Zip II (4)^a^			HD-Zip III (4)^a^			HD-Zip IV (4)^a^			Total - each species		
Species	O	E	R (%)	O	E	R (%)	O	E	R (%)	O	E	R (%)	O	E	R (%)
*Arabidopsis thaliana* (12)^b^	17	60	28.3	9	48	18.8	5	48	10.4	16	48	33.3	47	204	23.0
*Vitis vinifera* (3)^b^	14	15	93.3	7	12	58.3	4	12	33.3	8	12	66.7	33	51	64.7
*Glycine max* (12)^b^	36	60	60.0	24	48	50.0	11	48	22.9	30	48	62.5	101	204	49.5
*Medicago truncatula* (6)^b^	15	30	50.0	7	24	29.2	6	24	25.0	13	24	54.2	41	102	40.2
*Oryza sativa* (4)^b^	14	20	70.0	12	16	75.0	4	16	25.0	11	16	68.8	41	68	60.3
Total - Among five species	96	185	51.9	59	148	39.9	30	148	20.3	78	148	52.7	263	629	41.8

^a^Number of ancient angiosperm clades observed in each HD-Zip subfamily.

^b^Number of genes expected in each ancient angiosperm clade based on the history of whole genome duplication events.

In the phylogeny generated with sequences from eight species, the eudictos (poplar, cucumber, *Arabidopsis*, grape, soybean and *M. truncatula*) usually clustered together, with the monocots (rice and maize) as an outgroup (Additional file 5: Figure S5, Additional file 6: Figure S6, Additional file 7: Figure S7 and Additional file 8: Figure S8).

### Validation of HD-Zip genes using conserved domains, motifs and gene-structures

The HD-Zip I and II sequences contain the Homeobox (PF00046.24) domain and belong to the Homeobox associated leucine zipper family (HALZ; PF02183.13). In addition, the HD-Zip II sequences contain the conserved residues “CPSCE” at the carboxy terminal, and seven of the 24 HD-Zip II sequences contain a HD-ZIP_N (PF04618.7) domain at the N-terminal. The HD-Zip III sequences are highly conserved among all five species along the complete length of the coding sequence (Additional file 3: Figure S3). They contain the Homeobox (PF00046.24), START (PF01852.14) and MEKHLA (PF08670.6) domains. The HD-Zip IV sequences contain the Homeobox (PF00046.24) and the START (PF01852.14) domains. The presence of leucine zipper motif immediately following the homeodomain in HD-Zip III and IV sequences was confirmed using the sequence logos (Additional file 3: Figure S3, Additional file 4: Figure S4). Exon-intron structures are characteristic for each subfamily (Additional file 9: Figure S9, Additional file 10: Figure S10, Additional file 11: Figure S11 and Additional file 12: Figure S12). The HD-Zip III is particularly conserved, with each gene containing exactly 18 exons. The numbers of exons in genes in the HD-Zip I, II and IV subfamilies are in the ranges 1–5, 3–6, and 8–12. The HD-Zip I and II genes code for smaller proteins, with average peptide length of 265 and 275 amino acids, whereas HD-Zip III and IV genes code for average peptide lengths of 840 and 741 amino acids.

### Genomic locations of HD-Zip genes in the soybean genome

**Figure 5:**
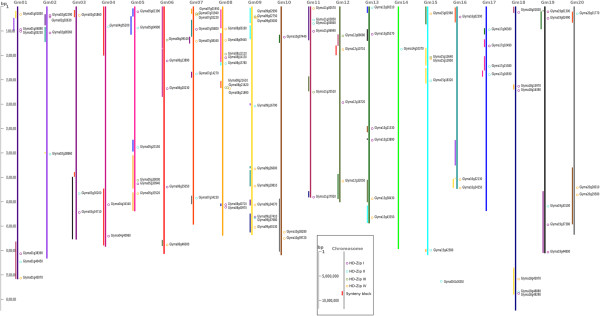
**Chromosomal locations and synteny relationships of soybean HD-Zip genes.** The chromosomal locations of the soybean HD-Zip genes were obtained from the GFF file of *Glycine max* assembly v1.01, annotation 1.0, and were displayed using chromosome visualization tool (CViT). All chromosomes and gene locations are shown to scale. Glyma0041s00350 located on scaffold 41 (149758–152298 bp) is included independently in the figure. The homoeologous gene pairs are identified with colored solid lines on the left side of the chromosomes. The chromosomes and the solid lines with identical colors are syntenic regions containing homoeologous genes. A detailed list of homoeologous HD-Zip genes is also provided in Additional file 13: Table S1.

The HD-Zip genes are distributed on all 20 chromosomes in the soybean genome, typically in the more gene-dense euchromatic regions near chromosome ends (Figure 5). One HD-Zip II gene (Glyma0041s00350) was found on an unanchored scaffold 41. The HD-Zip genes generally do not occur in clusters or arrays, with only three instances of tandemly duplicated genes.

### Genome duplications and expansion of HD-Zip family in the soybean genome

Copy number expansion of the HD-Zip family in the soybean genome has primarily occurred through genome duplication events (Figure 5, Additional file 13: Table S1). Each angiosperm clade in each of the four subfamilies (Figures 1, 2, 3 and 4) contains two to four soybean gene copies that are a result of retention of genes after the legume WGD (~59 Mya) and/or the *Glycine*-specific WGD (~13 Mya). Retention of genes following these WGDs has been high, with retention of 32 of 36 HD-Zip I (88.9%), 20 of 24 HD-Zip II (83.3%), 10 of 11 HD-Zip III (90.9), and 26 of 30 (86.7%) HD-Zip IV genes (Additional file 13: Table S1). There are two tandemly duplicated HD-Zip pairs in subfamily III, and another pair in subfamily IV. Phylogenetic patterns indicate that the tandemly duplicated genes in subfamily III further duplicated during a WGD event, giving rise to Glyma07g01940 and Glyma07g01950 on chromosome 07 and Glyma08g21610 and Glyma08g21620 on homoeologous chromosome 8. Genes Glyma09g02990 and Glyma09g03000, in HD-Zip IV, are another pair of tandemly duplicated genes. The Glycine WGD event resulted in the homoeologous gene pair Glyma09g03000 and Glyma15gg13950, whereas the homoeologous gene for Glyma09g02990 has evidently either been lost following the WGD – or the Glyma09g02990 and Glyma09g03000 duplication occurred after the Glycine WGD. Overall, 88 of the 101 HD-Zip genes are members of homoeologous gene pairs in the soybean genome.

### Expression of HD-Zip genes in 24 conditions including 17 tissues of soybean

**Figure 6:**
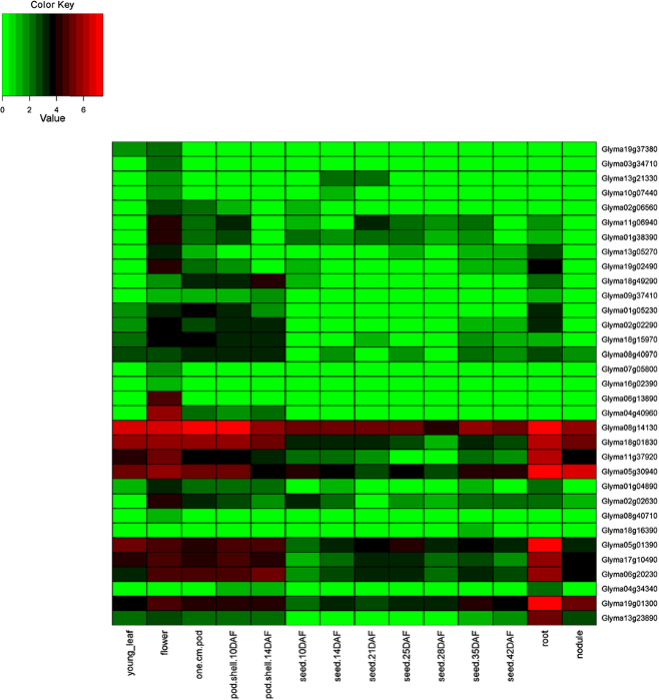
**Expression profiles of HD-Zip I genes in 14 tissues of soybean.** The Reads/Kb/Million (RPKM) normalized values of expressed genes was log_2_-transformed and visualized as heatmaps. Genes in the heatmap are ordered for consistency with the phylogeny in Figure 1. The abbreviation “DAF” in the tissue label represents “Days after flowering”.

**Figure 7:**
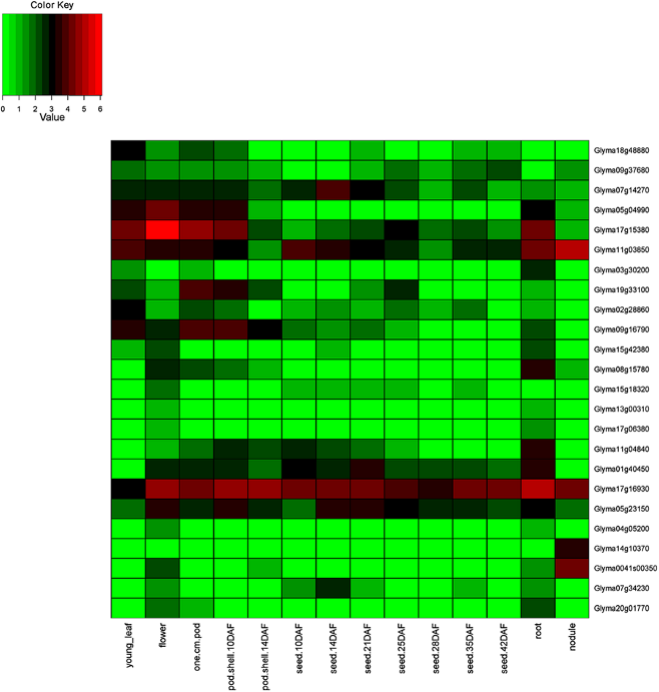
**Expression profiles of HD-Zip II genes in 14 tissues of soybean.** The Reads/Kb/Million (RPKM) normalized values of expressed genes was log_2_-transformed and visualized as heatmaps. Genes in the heatmap are ordered for consistency with the phylogeny in Figure 2. The abbreviation “DAF” in the tissue label represents “Days after flowering”.

**Figure 8:**
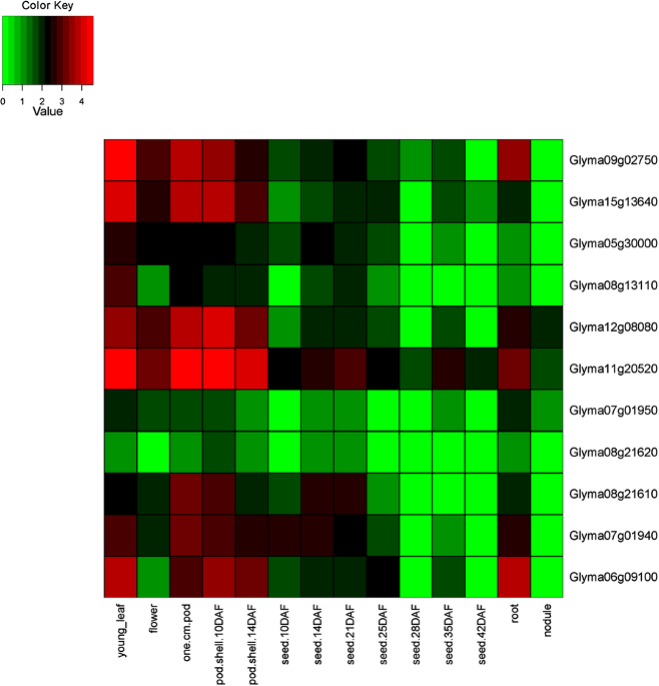
**Expression profiles of HD-Zip III genes in 14 tissues of soybean.** The Reads/Kb/Million (RPKM) normalized values of expressed genes was log_2_-transformed and visualized as heatmaps. Genes in the heatmap are ordered for consistency with the phylogeny in Figure 3. The abbreviation “DAF” in the tissue label represents “Days after flowering”.

**Figure 9:**
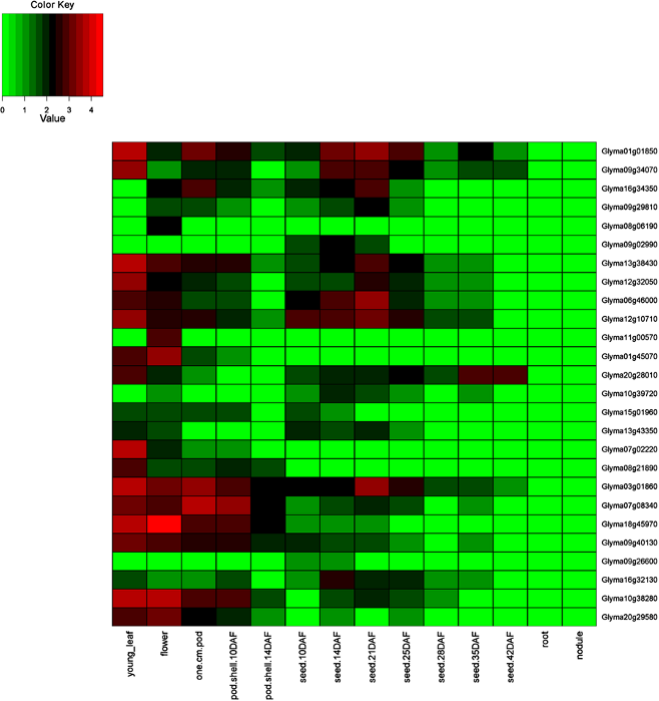
**Expression profiles of HD-Zip IV genes in 14 tissues of soybean.** The Reads/Kb/Million (RPKM) normalized values of expressed genes was log_2_-transformed and visualized as heatmaps. Genes in the heatmap are ordered for consistency with the phylogeny in Figure 4. The abbreviation “DAF” in the tissue label represents “Days after flowering”.

The expression of HD-Zip genes was investigated using the *G. max* gene expression atlas reported by Severin et al. [53], and Libault et al. [54, 55]. Of the 44 homoeologous gene pairs, 41 show expression in identical tissues (Figures 6, 7, 8, and 9, Additional file 14: Figure S13, Additional file 15: Figure S14, Additional file 16: Figure S15 and Additional file 17: Figure S16). The remaining three show divergent patterns in different tissues between the WGD-derived paralogs (Figures 6 and 9). HD-Zip I gene Glyma06g20230 was expressed in each of the 14 tissues, whereas the homoeolog Glyma04g34340 was expressed in the roots, “pod.shell.10DAF” and “pod.shell.14DAF”. HD-Zip I gene Glyma19g01300 had expression in each of the 14 tissues, but the homoeolog Glyma13g23890 lacked expression in five “seed tissues” (10 DAF, 14 DAF, 21 DAF, 25 DAF and 28 DAF). HD-Zip IV gene Glyma11g00570 was expressed only in the flower, whereas the homoeolog Glyma01g45070 was expressed in young leaf, flower, “one.cm.pod” and “pod.shell.10DAF”. Similar divergent gene expression patterns between these homoeologous genes were also noticed in the gene expression atlas reported by Libault et al. [54] (Additional file 14: Figure S13, Additional file 17: Figure S16).

Three HD-Zip I (Glyma12g18720, Glyma06g35050, and Glyma19g44800), and four HD-Zip IV (Glyma08g09440, Glyma15g13950, Glyma09g03000, and Glyma05g33520) genes showed no expression in any of the 14 tissues investigated by Severin et al. [53]. However, we found evidence of expression for Glyma12g18720 - HD-Zip I in the roots subjected to dehydration stress after 12 hr (data generated in this study). Glyma06g35050 - HD-Zip I showed expression in leaf, flower and root tip, whereas Glyma09g03000 and Glyma05g33520 - HD-Zip IV were expressed in green pods and shoot apical meristem respectively in Libault et al. [54]. The remaining three genes had no evidence for expression (Glyma19g44800 - HD-Zip I and Glyma08g09440, Glyma15g13950 - HD-Zip IV) in either of the two atlases. These three genes did not reveal any frame shift mutations when investigated at the sequence level. Hence, might be pseudogenes, or incorrectly predicted gene models – or they may only be expressed in certain tissues or under conditions that have not been sampled in this study.

Based on the mean expression of genes across 14 tissues investigated by Severin et al. [53], HD-Zip I and II genes had relatively higher expression in roots and flowers; HD-Zip III in young leaves, “one cm pod”, and “pod shell 10 days after flowering”; and HD-Zip IV in young leaves and flowers. Similar results were observed using the expression atlas generated by Libault et al. [54], with the exception of highest mean expression of genes belonging to each of the subfamilies was noticed in shoot apical meristem. Overall, HD-Zip genes had expression in each of the 17 tissues.

The screening of HD-Zip gene expression using mock-inoculated and *B. japonicum*-infected root hair cells at different time points highlighted HD-Zip genes with more than two fold expression differences between control and treatment samples (Additional file 18: Figure S17, Additional file 19: Figure S18, Additional file 20: Figure S19 and Additional file 21: Figure S20). More than 50% of the genes belonging to the HD-Zip III showed greater than two-fold difference between control and treatment samples at least at one time point (Additional file 20: Figure S19).

### Expression of HD-Zip genes under dehydration and salt stress using RNA-Seq

To identify HD-Zip family members responsive to abiotic stress, we used an RNA-seq approach. Twenty-one samples were analyzed by RNA-seq including three control samples (0 hr), and three biological replicates for each of the three time points 1, 6 and 12 hr under dehydration and salt stress. The total number of reads generated in the RNA-Seq experiment from sequencing of 21 sample libraries was 238.8 million, of which 181.2 million (75.9%) uniquely mapped to a single location in the soybean genome (Table 2).

**Table 2:** **Experimental set-up and summary of read-count data from RNA-Seq analysis**

Treatment	Time point (hr)	Replicate (#)	Lane on HiSeq 2000	Total reads	Uniquely mapped reads	Uniquely mapped reads (%)
Control	0	1	4	10,150,369	8,047,650	79.3%
Control	0	2	4	11,849,953	9,207,421	77.7%
Control	0	3	3	11,272,789	9,164,808	81.3%
Dehydration	1	1	3	6,875,669	5,571,406	81.0%
Dehydration	1	2	3	6,744,882	4,948,665	73.4%
Dehydration	1	3	3	8,650,675	6,612,402	76.4%
Dehydration	6	1	3	11,828,271	9,624,573	81.4%
Dehydration	6	2	3	11,355,361	8,599,941	75.7%
Dehydration	6	3	4	10,038,099	8,009,975	79.8%
Dehydration	12	1	4	9,270,260	7,194,931	77.6%
Dehydration	12	2	4	5,555,797	4,050,429	72.9%
Dehydration	12	3	4	5,105,827	4,087,108	80.0%
Salt	1	1	2	38,214,261	30,683,738	80.3%
Salt	1	2	2	9,046,880	7,428,387	82.1%
Salt	1	3	2	9,423,474	7,202,416	76.4%
Salt	6	1	2	7,445,356	5,580,211	74.9%
Salt	6	2	2	5,890,968	4,422,058	75.1%
Salt	6	3	2	25,296,306	14,687,424	58.1%
Salt	12	1	2	7,481,184	5,253,438	70.2%
Salt	12	2	3	14,201,579	11,170,436	78.7%
Salt	12	3	4	13,124,265	9,645,045	73.5%
			Total	238,822,225	181,192,462	75.9%
			Average	11,372,487	8,628,212	76.5%

We identified 4,389 and 8,077 genes to be DE in at least one of the three time points (1, 6 or 12 hr) under dehydration and salt stresses respectively (Additional file 22: Table S2, Additional file 23: Table S3, Additional file 24: Table S4, Additional file 25: Table S5, Additional file 26: Table S6 and Additional file 27: Table S7) (see Methods for the filtering criteria). Salt stress resulted in mostly upregulation of genes, whereas dehydration stress caused downregulation of genes (Additional file 28: Table S8). The number of genes discarded from the differential expression analysis due to significant amount of variation between the replicates under dehydration and salt stress at a given time point ranged from 119 to 220 (Additional file 28: Table S8). The raw and DESeq-normalized expression values for each gene model under both dehydration and salt stress at 1, 6 and 12 hr are provided in Additional file 29: Table S9 and Additional file 30: Table S10 respectively.

**Figure 10:**
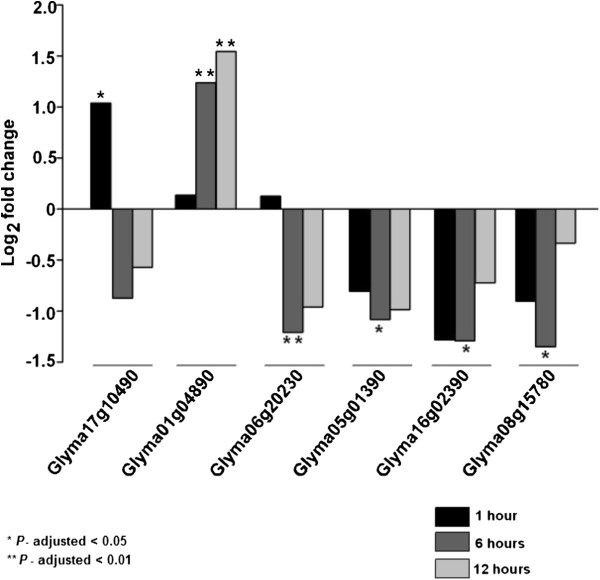
**RNA-Seq based expression profiles of HD-Zip genes that are differentially expressed in at least one time point under dehydration stress.** The HD-Zip genes responsive to dehydration stress at the first trifoliolate stage in the roots of soybean cv. Williams 82 at least at one time point (1, 6 or 12 hr) are shown. The criteria for differential expression includes, (1) *P*-value corrected for multiple testing correction using Benjamini and Hochberg [62] to be less than 0.05, (2) two folder or greater fold change, (3) residual variance quotients of both control and treatment samples be less than 20. The criterion (3) filters genes that have significant variation between replicates.

Six genes were DE at least at one of the three time points under dehydration stress (Figure 10): five in HD-Zip I, and one in HD-Zip II. Two genes were upregulated and the remaining four were downregulated under dehydration stress. Glyma01g04890 was significantly DE at two different time points. Three of the five DE HD-Zip I genes (Glyma17g10490, Glyma06g20230 and Glyma05g01390) belong to the angiosperm clade A5 (Figure 1), and were a result of the early-legume WGD and the recent *Glycine* WGD.

**Figure 11:**
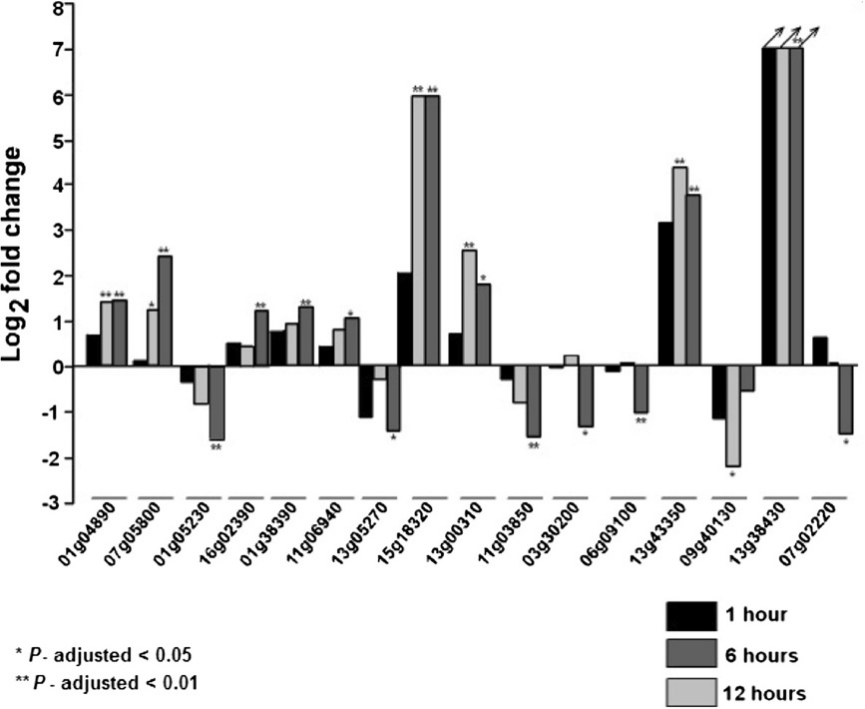
**RNA-Seq based expression profiles of HD-Zip genes that are differentially expressed in at least one time point under salt stress.** The HD-Zip genes responsive to salt stress at the first trifoliolate stage in the roots of soybean cv. Williams 82 at least at one time point (1, 6 or 12 hr) are shown. The criteria for differential expression includes, (1) *P*-value corrected for multiple testing correction using Benjamini and Hochberg [62] to be less than 0.05, (2) two folder or greater fold change, (3) residual variance quotients of both control and treatment samples be less than 20. The criterion (3) filters genes that have significant variation between replicates.

We found sixteen genes DE at one of the three time points under salt stress (Figure 11): seven in HD-Zip I, four in HD-Zip II, one in HD-Zip III, and four in HD-Zip IV. Nine genes were upregulated and the remaining seven genes were downregulated under salt stress. Five of the 16 genes were significantly DE at two time points (HD-Zip I: Glyma01g04890, Glyma07g05800; HD-Zip II: Glyma15g18320, Glyma13g00310; HD-Zip IV: Glyma13g43350). Four of the seven DE HD-Zip I genes were two homoeologous gene pairs (Glyma07g05800/Glyma16g02390; Glyma01g38390/Glyma11g06940). One of the pairs is a member of angiosperm clade A3, and the other belongs to angiosperm clade A1 (Figure 1). One pair each from the HD-Zip II and HD-Zip IV DE genes (Glyma15g18320/Glyma13g00310 and Glyma13g43350/Glyma07g02220, respectively) resulted from the early-legume WGD. The HD-Zip IV gene Glyma13g38430 was not expressed under the control condition (0 hr time point), but was upregulated after 12 hr under salt stress.

The two HD-Zip I genes, Glyma01g04890 and Glyma16g02390, were DE under both dehydration and salt stress. Glyma01g04890 was upregulated at the 6 hr and 12 hr time points under both stress treatments, whereas Glyma16g02390 was downregulated at the 6 hr time point under dehydration stress, and upregulated at the 12 hr time point under salt stress. In summary, 20 of the 101 HD-Zip genes in soybean were DE under either dehydration or salt stress, at least at one time point. Eleven of these 20 genes shared a common ancestor either before the early-legume or the *Glycine* WGDs, implying conservation of gene functions following these genome duplications.

### Annotation of differentially expressed genes under dehydration and salt stress

**Figure 12:**
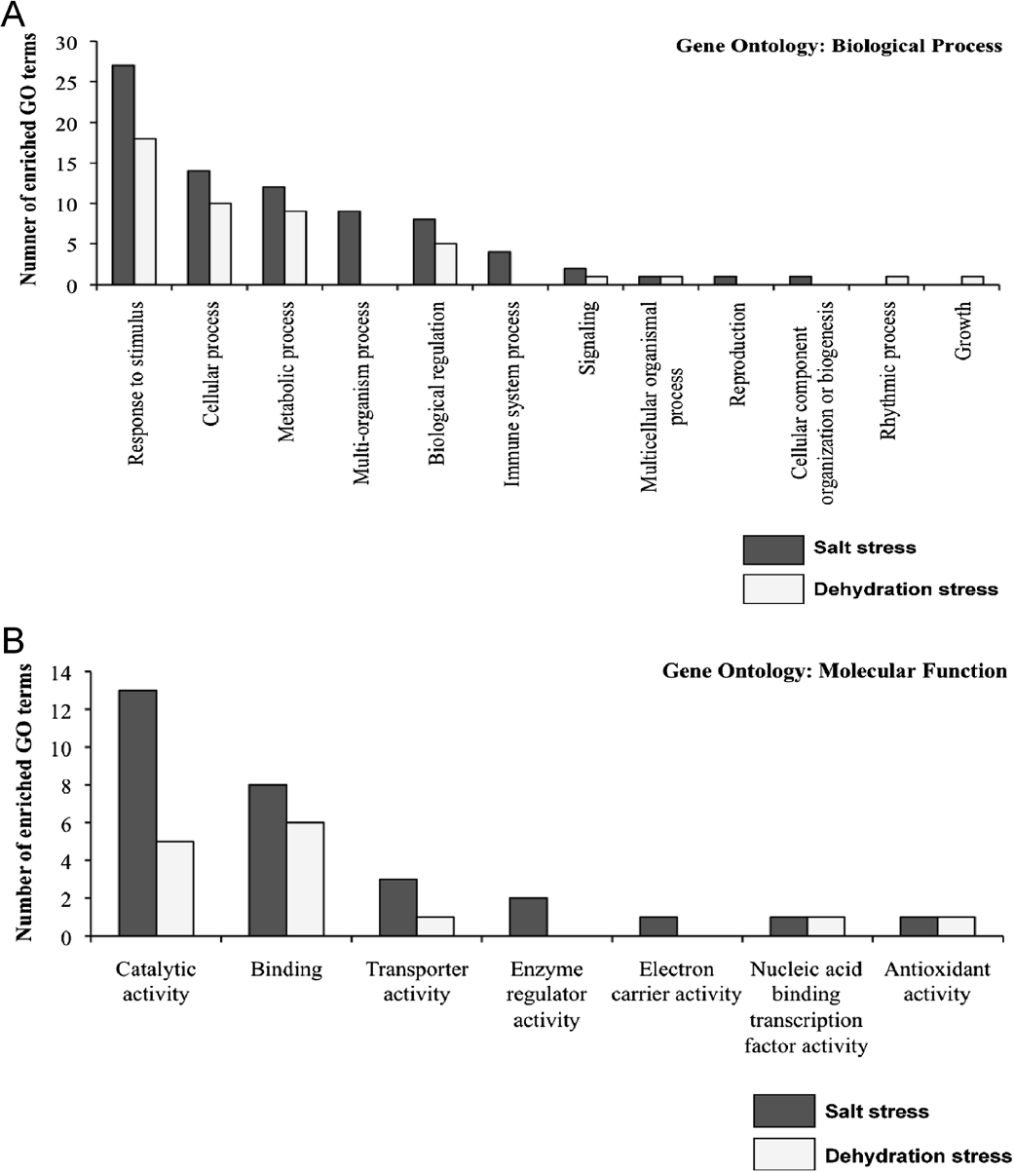
**Gene ontology biological process (A) and molecular function (B) categories significantly (corrected** ***P*** **<0.05) overrepresented among differentially expressed genes under dehydration and salt stress.** Differentially expressed genes under dehydration and salt stress were annotated using the top *Arabidopsis* hit, and then screened for overrepresented GO terms against all soybean genes using Fisher’s exact test [66] and Bonferroni [67] corrected significance value of less than 0.05 (Additional file 31: Table S11). The overrepresented GO terms were enriched at the second level using BLAST2GO v.2.7.1 [68] and are shown in the figure.

In order to help evaluate and confirm results from the application of dehydration and salt stress treatments, GO and TF enrichment analysis were performed on the DE genes. Under dehydration stress, 28 “biological process” and 15 “molecular function” terms were significantly (corrected *P* <0.05) overrepresented, whereas 41 “biological process” and 27 “molecular function” terms were significantly (corrected *P* <0.05) overrepresented under salt stress (Additional file 31: Table S11). The enriched biological processes and molecular functions include terms such as - “GO:0009414 - response to water deprivation”, “GO:0015250 - water channel activity”, and “GO:0009651 - response to salt stress”, consistent with the experimental treatments (dehydration and salt stress). At the second level of GO analysis, the biological process category “response to stimulus” was the most prevalent one under both stress treatments, followed by “cellular process” and “metabolic process” (Figure 12A), while in the molecular function category, “catalytic activity” and “binding” were highly represented (Figure 12B).

We identified 503 and 862 TFs among the DE genes under dehydration and salt stress treatments respectively (Additional file 32: Table S12). These TFs corresponded to 35 and 47 TF classes under dehydration and salt stress. Using the enrichment analysis, we identified four TF classes, “WRKY”, “AP2-EREBP”, “ZIM” and “C2C2 (Zn) CO-like” to be significantly (corrected *P* <0.05) overrepresented under both stress treatments, whereas the TF class “NAC” was overrepresented only under salt stress (Table 3).

**Table 3:** **Transcription factor class significantly (corrected** ***P*** **<0.05) overrepresented among the differentially expressed genes under dehydration and salt stress**

Transcription factor class	Genome count	Salt stress expression count	Corrected ***P***-value	Dehydration stress expression count	Corrected ***P***-value	Role in abiotic stress response
WRKY	197	82	2.52E-21	34	3.45E-03	[90–92]
AP2-EREBP	381	111	1.67E-14	75	2.88E-10	[93–95]
ZIM	24	20	9.55E-13	16	1.28E-10	[96–98]
C2C2 (Zn) CO-like	72	33	9.94E-10	26	3.77E-09	[99–101]
NAC	208	49	3.48E-03	NA	NA	[102–104]

### Promoter analysis

The enrichment analysis performed with the Clover program [71] and the TRANSFAC database [72] on the promoters of HD-Zip genes identified four different transcription factor binding sites (TFBSs) overrepresented in the promoters of HD-Zip I genes, and at least 9 different TFBSs in HD-Zip II to IV genes (Table 4). The genes belonging to the same subfamily had a diverse profile of TFBSs enriched in the promoters, suggesting the possible role of promoter sequences in functional diversification of the HD-Zip genes of the same subfamilies (Additional file 33: Table S13). The homoeologous genes in all subfamilies had reasonably different TFBSs enriched in their promoters, suggesting specific regulation of homoeologous genes under particular conditions (Additional file 33: Table S13).

**Table 4:** **Plant transcription factor binding sites significantly (** ***P*** **<0.05) overrepresented in the promoters of HD-Zip genes belonging to each of the subfamilies**

	Motif #	^1^TFBS	^2^Count	^3^Proportion	^4^TFBS_Dehydration	^5^TFBS_Salt	^6^TF_Class	^7^DE_Dehydration	^8^DE_Salt
HD-Zip I	M00354	Dof3	33	91.7	-	-	Dof	+	+
	M00700	ROM	31	86.1	+	-	bZIP	+	+
	M01136	Dof	29	80.6	-	-	Dof	+	+
	M00353	Dof2	27	75.0	-	-	Dof	+	+
^9^HD-Zip II	M00479	Alfin1	21	91.3	-	-	PHD	+	+
	M01136	Dof	20	87.0	-	-	Dof	+	+
	M00354	Dof3	19	82.6	-	-	Dof	+	+
	M00440	CG1	18	78.3	-	-	CAMTA	+	+
	M00506	LIM1	18	78.3	-	-	LIM	-	+
	M00502	TEIL	17	73.9	-	+	^10^AP2-EREBP	+	+
	M00653	OCSBF-1	17	73.9	+	+	bZIP	+	+
	M00788	EmBP-1b	17	73.9	+	+	bZIP	+	+
	M01128	SED	17	73.9	-	-	DOF	+	+
	M00942	CPRF-1	16	69.6	+	-	bZIP	+	+
	M00948	PCF2	16	69.6	+	-	TCP	+	+
	M00443	Opaque-2	14	60.9	+	+	bZIP	+	+
	M01133	AG	14	60.9	-	-	MADS	+	+
	M00660	RITA-1	13	56.5	+	+	bZIP	+	+
	M01130	PBF	13	56.5	-	-	Dof	+	+
	M01054	bHLH66	12	52.2	+	+	bHLH	+	+
	M00503	ATHB-5	11	47.8	+	-	HD-Zip I	+	+
	M00434	PIF3	10	43.5	+	+	bHLH	+	+
HD-Zip III	M00479	Alfin1	10	90.9	-	-	PHD	+	+
	M00438	ARF	9	81.8	-	-	ARF	+	+
	M01021	ID1	9	81.8	+	-	C2H2 - zinc	+	+
	M01126	BPC1	8	72.7	-	-	BBR/BPC	-	-
	M00948	PCF2	7	63.6	+	-	TCP	+	+
	^11^M00151	AG	7	63.6	-	-	MADS	+	+
	M00820	HAHB-4	6	54.5	-	-	HD-Zip I	+	+
	^12^M01061	AGL2	6	54.5	-	-	MADS	+	+
	M00392	AGL3	5	45.5	-	-	MADS	+	+
	M00949	AGL15	5	45.5	-	-	MADS	+	+
HD-Zip IV	^13^M00355	PBF	29	96.7	+	-	Dof	+	+
	M00438	ARF	25	83.3	-	-	ARF	+	+
	M01126	BPC1	25	83.3	-	-	BBR/BPC	-	-
	M01136	Dof	25	83.3	-	-	Dof	+	+
	M01128	SED	23	76.7	-	-	DOF	+	+
	M01021	ID1	22	73.3	+	-	C2H2 - zinc	+	+
	M00702	SPF1	20	66.7	-	+	^10^WRKY	+	+
	M00654	OSBZ8	15	50.0	+	+	bZIP	+	+
	M00089	Athb-1	11	36.7	+	-	HD-Zip I	+	+

^1^TFBS: Transcription factor binding site (TFBS) significantly (*P* <0.05, motif score >5) overrepresented in the promoters of HD-Zip genes.

^2^Count: Number of HD-Zip genes within a subfamily that contain the TFBS significantly overrepresented in their promoters.

^3^Proportion: Percentage of HD-Zip genes within a subfamily that contain the TFBS overrepresented in their promoters.

^4^TFBS_Dehydration: " + " indicates that the respective TFBS is overrepresented in the promoters of genes that were differentially expressed under dehydration stress, and "-" represents not overrepresented.

^5^TFBS_Salt: " + " indicates that the respective TFBS is overrepresented in the promoters of genes that were differentially expressed under salt stress, and "-" represents not overrepresented.

^6^TF_Class: The membership of TFBS to a particular transcription factor (TF) class based on TRANSFAC [72] and UniprotKB [134].

^7^DE_Dehydration: " + " indicates members of the respective TF class are differentially expressed (DE) under dehydration stress, and “-” indicates otherwise.

^8^DE_Salt: " + " indicates members of the respective TF class are DE under salt stress, and “-” indicates otherwise.

^9^Although the HD-Zip II subfamily has 24 genes, the proportion is calculated using 23 genes. HD-Zip II gene Glyma05g23150 was excluded from the promoter analysis due to the selection criteria utilized (see methods for promoter selection criteria).

^10^TF class significantly (corrected *P* <0.05) overrepresented in the DE genes under dehydration and salt stress.

^11^AG TFBS has multiple motif identifiers - M00151, M01063, M01133, and M00950. Counts of AG TFBS’s irrespective of the identifier# were summed to estimate total count and proportion.

^12^AGL2 TFBS has two motif identifiers - M01061 and M01062. Counts of AGL2 TFBS’s irrespective of the identifier# were summed to estimate total count and proportion.

^13^PBF TFBS has two motif identifiers - M00355 and M01130. Counts of PBF TFBS’s irrespective of the identifier# were summed to estimate total count and proportion.

There are 14 TFBSs overrepresented in the promoters of HD-Zip genes as well as promoters of DE genes under dehydration stress. Similarly nine TFBSs are overrepresented in the promoters of HD-Zip genes and the promoters of DE genes under salt stress (Table 4, Additional file 34: Table S14). These TF classes are potential candidates that may influence both HD-Zip genes as well genes involved in dehydration and salt stress responses.

The TFBSs “Dof3” and “PBF” are overrepresented in more than 90% of the HD-Zip I and IV genes respectively, and “Alfin1” is overrepresented in more than 90% of HD-Zip II and III genes (Table 4). Hence, these transcription factors probably play an important role in regulating certain HD-Zip genes.

Finally, all but three TF classes corresponding to enriched TFBSs in the promoters of HD-Zip genes contain DE genes under dehydration and salt stress (Table 4). This observation is consistent with HD-Zip genes playing important roles under dehydration and salt stress-responses.

## Discussion

### Identification and phylogenetic analysis of HD-Zip genes

In this study we have identified and characterized 101 HD-Zip genes in the soybean genome. Recently, 88 HD-Zip genes have been described in soybean [12]. Chen et al. [12] used BLASTP to identify 100 putative HD-Zip transcription factors. SMART and PFAM analyses requiring both an HD and LZ domain were used to refine the number of HD-Zip genes to 88. Similarly, we initiated our study using BLASTP of Arabidopsis HD-Zip genes against the proteomes of soybean, *M. truncatula*, rice and grape. We then used phylogenetic analyses coupled with HMM searches, domain analyses, and known evolutionary relationships among the five species, to identify more diverse members of the HD-Zip family in each of these species. Using this approach, we were able to identify 13 additional novel HD-Zip genes in soybean and identify the HD-Zips in *M. truncatula* and grape, which had previously been unreported. Not surprisingly, our approach had the biggest impact on the largely uncharacterized HD-Zip IV genes. While Chen et al. [12] reported 19 genes in HD-Zip IV, we have found 30 genes. These genes may have novel biological functions.

By including multiple species in our search for HD-Zip genes, we also improved the classification of the different family members in soybean and other species. The clustering of *Arabidopsis* genes in the HD-Zip subfamilies was consistent with the results of Ariel et al. [1]. The HD-Zip I and II subfamilies can be classified into nine (α, β1, β2, γ, δ, ϵ, ϕ1, ϕ2 and ζ) and four (α, β, γ and δ) clades respectively that have been previously described in studies on *Arabidopsis*, rice and maize [4, 7, 8] (data not shown). Although the results in our study are consistent with the later classification, we suggest that the later strategy be used with discretion. One instance where it can lead to conflicting results is that the ζ clade has been described as monocot-specific clade in all previous studies [4, 7, 8], but this clade clearly contained dicot sequences as a part of an old angiosperm clade in our study. One potential reason for this conflict is that the previous studies included only *Arabidopsis* [4, 8] or *Arabidopsis* and *C. plantagineum* [7] as the dicot species. Sampling of additional dicot sequences of soybean, *M. truncatula* and grape in this study provided a clearer picture of the taxonomic contexts of the HD-Zip gene family.

We identified five ancient angiosperm clades in HD-Zip I, and four in the HD-Zip II, III and IV subfamilies. The presence of these multiple angiosperm clades in each subfamily is consistent with the recent discovery of two ancient WGD events, one occurring at the base of the angiosperm lineage (ancient angiosperm WGD) and the other before the angiosperm-gymnosperm split (ancestral seed plant WGD) [31]. Early diversification driven by multiple early-plant WGDs is also consistent with a previous study of the evolution of HD-Zip III subfamily in land plants [73]. The presence of five angiosperm clades in HD-Zip I (rather than the four that would be expected from two early WGDs) is intriguing and needs further investigation in the context of synteny analysis and inclusion of additional species in the phylogeny.

A phylogeny with eight species, including published HD-Zip sequences from maize, poplar and cucumber, was largely congruent with the phylogeny generated using five species. These phylogenetic relationships will help identify orthologous genes, and accelerate functional characterizations studies.

### Conserved domains and gene structures for validation of HD-Zip genes

PFAM and sequence logos identify highly conserved domains and motifs in the HD-Zip gene family. These have been reported in previous studies [1, 2, 4, 12], but we note two exceptions: *Arabidopsis* HD-Zip I gene AT1G27050 had an additional “RRM_1” (RNA recognition motif), and *Medicago* HD-Zip IV gene Mt.ctg127898_1 had two START domains. Overall, the highly conserved domains and motifs are the signatures of the HD-Zip gene family and can be utilized to validate genes identified using several approaches.

Exon-intron structures are generally well conserved in each HD-Zip subfamily, particularly within each angiosperm clade. The HD-Zip III gene-structures were remarkably conserved, with each of the soybean genes having precisely 18 exons. Considering HD-Zip III gene structures reported in other species, all genes in poplar had 18 exons [9], and 4 of the 5 maize genes had 18 exons [8], but in rice only one of the four genes had 18 exons [7]. The generally well-conserved exon structure in HD-Zip III genes across different species highlights the possibility of conserved gene function and strict regulation of these genes. In a recent study involving identification of genes that are potential targets of miRNA in developing soybean seeds, all HD-Zip III genes were found to be targets of miRNA 166 [74]. Prigge and Clark [73], and Floyd and Bowman [75] have previously suggested that HD-Zip III sequences across all land plants produce transcripts that could be targeted by miRNA165 and miRNA166. DeRocher and Nguyen [76] overexpressed *Arabidopsis* HD-Zip III gene REVOLUTA in soybean embryo, leading to seed yield increase with no change in the seed composition. In short, the HD-Zip III genes appear to be both highly conserved and under intricate transcriptional regulation.

### Expansion of HD-Zip gene family

The 101 HD-Zip genes in soybean is the highest number reported so far in any angiosperm species, comparing with 48 in *Arabidopsis* [2], 55 in maize [8], 47 in rice [2], and 63 in poplar [9]. The HD-Zip genes in soybean have expanded during the early-legume WGD event (~59 Mya), and the *Glycine* WGD event (~13 Mya), with high retention of paralogs. Expansion of the HD-Zip gene family due to WGDs has been previously reported in other species. The *Arabidopsis*, rice, maize and poplar have at least 75%, 50%, 62% and 81% homoeologous gene pairs respectively [5, 6, 8, 9, 77–79]. However, in cucumber, a species that lacks WGD events since eudicot radiation, there are no homoeologous gene pairs among HD-Zip I and IV (the two subfamilies described in cucumber) [10, 11]. These results imply that the HD-Zip gene family has expanded in a species-specific manner, with copy number generally depending on WGD events and high retention rates after duplications.

Gene families can be broadly categorized as having high rates of retention of segmental (WGD-derived) duplicates and low generation or retention of tandem duplicates – or vice versa (low segmental retention, high tandem generation and retention) [80]. The low-tandem/high-segmental duplication class of gene families has been reported to comprise highly conserved, housekeeping, and key regulatory gene families [80] – for example, transcription factor families such as heat shock and WRKY, housekeeping families such as mitochondrial carrier proteins [81, 82], and the proteasome 20S subunit family [83, 84]. Clearly, the HD-Zip superfamily falls in the “high segmental, low tandem” category, with only three tandem duplication events in the HD-Zip genes in soybean. The expansion and retention of the HD-Zip family during segmental duplication events will have consequences for functional characterization studies, due to the possibility of genetic redundancy in duplicated genes.

### Gene expression patterns of HD-Zip genes in 24 conditions, including 17 tissues

The *G. max* expression atlas [53] was initially utilized for investigating gene expression patterns of HD-Zip genes in 14 tissues of soybean. The average expression values across 14 tissues for each subfamily was highly variable, and there were genes with extremely high expression relative to the average expression across tissues in each of the subfamily. Investigating gene expression patterns separately for each subfamily on a log_2_-transformed scale helped identify gene expression patterns that were unreported in Chen et al. [12]. Chen et al. [12] displayed expression of all four subfamilies on a single scale using average linkage clustering method. In addition we utilized two additional gene expression atlases developed by Libault et al. [54, 55], which allowed investigation of HD-Zip genes in three additional tissues, and seven different conditions.

All but three homoeologous gene pairs show consistent expression in the same tissues between the WGD-derived paralogs, suggesting retention of HD-Zip gene functions after genome duplications. The genome duplication events provide raw materials for new gene functions. The duplicated gene can evolve to have a new function (neofunctionalization) [85] or can acquire new deleterious mutations and become a pseudogene (pseudogneization); or both the ancestral and the newly formed gene can undergo reduction in their levels and patterns of activity, such that jointly their function matches with that of the ancestral gene (subfunctionalization) [86].

### RNA-Seq based expression profiling of soybean genes during dehydration and salt stress

RNA-Seq analysis was utilized to investigate genes involved in dehydration and salt stress. The expression of all soybean genes including the 101 HD-Zip genes identified in this study was studied in the roots of soybean cv. Williams 82 at V1 stage, at four different time points, and under dehydration and salt stress. The evaluation of plants at the V1 stage may assist in identification of candidate genes involved in initiation of dehydration and salt stress. Recently, Chen et al. [12] reported the influence of drought and salinity stress on HD-Zip genes using publicly available microarray data sets available at National Center for Biotechnology Information under accession numbers GSE41125 and GSE40627. The microarray datasets facilitated investigation of the expression of 55 of the 88 HD-Zip genes identified in their study. The microarray dataset GSE40627 reports expression of genes in the leaves under drought stress imposed at late developmental stages (V6 and R2), whereas the dataset GSE41125 describes expression of genes in 14 d seedlings utilizing pooled RNA samples from 0, 3, 6, 12 and 24 hr of mock and salinity stressed plants. Thus, in the current study, the utilization of root tissue at the V1 stage, and investigation of gene expression separately at each of the four time points 0, 1, 6 and 12 hr provided clearly different and more precise insight into genes that are involved in dehydration and salt stress.

We identified 4,389 and 8,077 genes to be DE in the roots of soybean cv. Williams 82 at the V1 stage at least at one of the three time points (1, 6 or 12 hr) under dehydration and salt stress respectively. Partial validation of DE genes for their role in abiotic stress responses was obtained by performing GO and TF enrichment analysis. The highly represented biological process GO categories, “response to stimulus”, “cellular process”, and “metabolic process” as well as the molecular function categories, “catalytic activity” and “binding”, are generally found to be enriched during abiotic stress responses [87–89]. Similarly, the four TF classes WRKY [90–92], AP2-EREBP [93–95], ZIM [96–98] and C2C2 (Zn) CO-like [99–101] (all enriched under both dehydration and salt stresses), and NAC [102–104] (overrepresented under salt stress) are major TFs that have previously been shown to play critical role in stress responses, and are consistent with results reported in this study.

### Expression profiling of HD-Zip genes under dehydration stress

RNA-Seq analysis identified 20 HD-Zip genes DE in the roots of soybean cv. “Williams 82”, under dehydration and salt stress. The role of HD-Zip genes in regulation of developmental adaptation under different environmental stress conditions has been previously established in *Arabidopsis*, *Medicago*, rice, sunflower, maize, cucumber, and poplar [4, 7–11, 19, 105–108].

All six genes identified as DE in the roots under dehydration stress in this study, were also, DE under drought stress in leaves [12]. Four of the five DE HD-Zip I genes belong to the angiosperm clade A5. This clade contains genes such as *CPHB-5* from *C. plantagineum*, and *Zmhdz1*, −*2*, −*3* from maize, that have previously been shown to have a role in water-stress response [4, 8, 20]. Chen et al. [109] showed Glyma06g20230 DE in this study was DE under dehydration stress, in the roots of drought-tolerant soybean genotype, “Jindou21”.

HD-Zip I gene Glyma16g02390 that is DE under dehydration stress belongs to the angiosperm clade A3. Genes in this clade have been extensively characterized for their role in water-stress responses in other species. For example, the *Arabidopsis* ATHB7 and ATHB12 genes have been shown to reduce plant growth under water-deficit condition [13, 16, 17]. The sunflower *HaHB4* gene is strongly induced by water deficit stress [14], and when over-expressed in *Arabidopsis* the plants exhibit increased survival by a process that inhibits-drought related senescence [18, 19]. The *N. attenuata NaHD20* gene is induced in roots under water-deficit conditions [15]. The rice *Oshox6*, *22* and *24* genes are involved in drought-responsiveness [7]. Hence, we hypothesize that the soybean gene Glyma16g02390 may have a role under water-deficit stress response and is a potential candidate for functional characterization.

HD-Zip II gene Glyma08g15780 that is DE under dehydration stress is an ortholog of rice genes *Oshox11* and *Oshox27*, which have also been demonstrated to be involved in drought-response [7].

In summary, the HD-Zip I and II genes show differential expression patterns under dehydration stress that are consistent with the water-deficit stress response functions of orthologous genes previously identified in studies of water stress. These results support that HD-Zip I and II genes may generally have a role, conserved across many angiosperm species, in mediating water-stress responses; and that these genes may be viable targets for developing more drought-tolerant soybean cultivars.

### Expression profiling of HD-Zip genes under salt stress

A subset of HD-Zip genes, from each of the four subfamilies, responded to salt (100 mM NaCl) stress in the roots, in at least one of the three time points. Six of the 16 genes (Glyma01g04890, Glyma07g05800, Glyma16g02390, Glyma13g05270, Glyma15g18320, Glyma03g30200) DE under salt stress have been recently shown to respond to salt stress, in 14 d old seedlings of soybean plant, in a microarray experiment [12].

The HD-Zip I gene Glyma13g05270 was downregulated under salt stress, which is similar to the expression of its Arabidopsis orthologs, *ATHB3* and *ATHB20*, which are similarly downregulated under salt stress [5]. The homoeologous genes Glyma01g38390 and Glyma11g06940 were upregulated after 12 hr of salt stress, comparable to the *Arabidopsis* orthologs, *ATHB20*, *ATHB50* and *ATHB53*, which are upregulated more than two-fold under salt stress [5].

Two of the four DE HD-Zip IV genes, Glyma13g43350 and Glyma13g38430, had nearly zero expression under control conditions, but were upregulated under salt stress, suggesting a possible role in root development under stress conditions. Glyma1343350 and Glyma07g02220 are orthologs of the *Arabidopsis* gene GLABRA2, which has been functionally characterized and shown to regulate root hair development, and cell specification of root epidermis in salt stressed plants [78, 110–112].

The two homoeologous HD-Zip I genes (Glyma07g05800 and Glyma16g02390) upregulated under salt stress belong to the angiosperm clade “A3”. This clade contains the functionally characterized *Medicago* gene MtHB1 (Medtr8g026960). *MtHB1* is induced in the roots under ABA and salt stress, and regulates lateral root emergence in *Medicago* [26]. The reduction of lateral root emergence by MtHB1, under salt stress, is a mechanism to minimize the exposure of plant roots to excess salt in the soil.

The HD-Zip I gene Glyma01g04890 was upregulated at 6 and 12 hr time points under both salt and dehydration stress. This gene was also upregulated under both drought and salt stress in the leaves and seedlings, respectively, in two microarray experiments [12]. A BLASTP search with Glyma01g04890 protein sequence against the patent database [113, 114] found a match (E-value = 0; Similarity >99.4%; Coverage = 100%) with sequences in five “patent applications” (US_2012_0278947_A1; US_2012_0096584_A1; US_2007_0277269_A1; US_2012_0005773_A1; US_2009_0144847_A1) that described the role of this sequence in improving plant performance under abiotic stress.

### Functional diversity and regulation of HD-Zip genes

The presence of highly diverse TFBSs enriched in the promoters of HD-Zip genes provides evidence for functional diversity. Previous studies have mainly focused on HD-Zip target-sequences, and regulatory regions adjacent to the DNA-binding domain of HD-Zip genes. All experimentally tested HD-Zip I genes have been shown to bind specifically, and with high affinity to target-sequences comprising of the same pseudopalindromic sequence CAAT(A/T)ATTG, under *in vitro* conditions [115–117]. Arce et al. [118] reported the presence of activation domain, sumoylation, and phosphorylation sites in the carboxy-terminal regions, and some putative regulatory regions in the amino-terminal regions, as being responsible for the functional diversity of HD-Zip I genes.

The “Dof3” and “PBF” TFBSs are enriched in more than 90% of HD-Zip I and IV gene promoters respectively. The “Dof” TFs like HD-Zip are plant-specific TFs and are involved in several process, for example stress-responses [119–121], phytochrome signaling [122], light-responses [123, 124], responses to plant hormones including auxin [125, 126] and gibberellin [127, 128], and seed germination [129, 130]. PBF also known as whirly family are known to regulate plant defense gene expression [131].

The TFBS “Alfin1” is overrepresented in more than 90% of HD-Zip II and III gene promoters. “Alfin1” TFs are shown to contribute toward salt tolerance in plants [132, 133].

Finally, the presence of highly diverse TFBSs enriched in the promoters of HD-Zip genes, both within and across subfamilies, suggests the complex integration of HD-Zip genes in various signal-transduction pathways, with a potential source for functional diversity of these highly conserved HD-Zip genes.

## Conclusions

In this study we have described the soybean HD-Zip gene superfamily. Evolutionary histories, interpreted in the context of whole genome duplication events and analysis of gene structures, provide additional verification for the classification of the soybean HD-Zip genes. The HD-Zip genes in the soybean genome were preferentially retained after the legume-specific and/or *Glycine*-specific whole genome duplication events. The RNA-Seq experiment identified candidate genes that may be involved in dehydration and salt stress responses.

## Electronic supplementary material

Additional file 1: Figure S1: Sequence logo of HD-Zip I displaying the conserved residues in HMM alignment. (TIFF 246 KB)

Additional file 2: Figure S2: Sequence logo of HD-Zip II displaying the conserved residues in HMM alignment. (TIFF 525 KB)

Additional file 3: Figure S3: Sequence logo of HD-Zip III displaying the conserved residues in HMM alignment. (TIFF 5 MB)

Additional file 4: Figure S4: Sequence logo of HD-Zip IV displaying the conserved residues in HMM alignment. (TIFF 3 MB)

Additional file 5: Figure S5: Phylogenetic relationships of HD-Zip I proteins from soybean, *Medicago*, *Arabidopsis*, grape, poplar, cucumber, maize and rice. The phylogenetic tree was built using the maximum likelihood method implemented in PhyML. The letters A1- A5 represent ancient angiosperm clades, based on whole genome duplication events, and the copy number of genes from each of the species. The letters are ordered for consistency with the phylogeny in Figure 1. The branch support values estimated using approximate likelihood ratio test (aLRT) are displayed in percentages. Rooting of the tree was inferred from Ariel et al. [1], angiosperm clade composition, and outgroup sequences from other subfamilies. (TIFF 1 MB)

Additional file 6: Figure S6: Phylogenetic relationships of HD-Zip II proteins from soybean, *Medicago*, *Arabidopsis*, grape, poplar, maize and rice. The phylogenetic tree was built using the maximum likelihood method implemented in PhyML. The letters A1- A4 represent ancient angiosperm clades, based on whole genome duplication events, and the copy number of genes from each of the species. The letters are ordered for consistency with the phylogeny in Figure 2. The branch support values estimated using approximate likelihood ratio test (aLRT) are displayed in percentages. Rooting of the tree was inferred from Ariel et al. [1], angiosperm clade composition, and outgroup sequences from other subfamilies. (TIFF 994 KB)

Additional file 7: Figure S7: Phylogenetic relationships of HD-Zip III proteins from soybean, *Medicago*, *Arabidopsis*, grape, poplar, maize and rice. The phylogenetic tree was built using the maximum likelihood method implemented in PhyML. The letters A1- A4 represent ancient angiosperm clades, based on whole genome duplication events, and the copy number of genes from each of the species. The letters are ordered for consistency with the phylogeny in Figure 3. The branch support values estimated using approximate likelihood ratio test (aLRT) are displayed in percentages. Rooting of the tree was inferred from Ariel et al. [1], angiosperm clade composition, and outgroup sequences from other subfamilies. (TIFF 549 KB)

Additional file 8: Figure S8: Phylogenetic relationships of HD-Zip IV proteins from soybean, *Medicago*, *Arabidopsis*, grape, poplar, cucumber, maize and rice. The phylogenetic tree was built using the maximum likelihood method implemented in PhyML. The letters A1- A4 represent ancient angiosperm clades, based on whole genome duplication events, and the copy number of genes from each of the species. The letters are ordered for consistency with the phylogeny in Figure 4. The branch support values estimated using approximate likelihood ratio test (aLRT) are displayed in percentages. Rooting of the tree was inferred from Ariel et al. [1], angiosperm clade composition, and outgroup sequences from other subfamilies. Genes Medtr5g005600.1 and Os01g57890 belong to the angiosperm clade “A2”. These two genes are not shown in the phylogeny because adding them significantly affects the topology. (TIFF 1 MB)

Additional file 9: Figure S9: Gene structure of HD-Zip I genes showing the exon-intron structure. (TIFF 676 KB)

Additional file 10: Figure S10: Gene structure of HD-Zip II genes showing the exon-intron structure. (TIFF 193 KB)

Additional file 11: Figure S11: Gene structure of HD-Zip III genes showing the exon-intron structure. (TIFF 114 KB)

Additional file 12: Figure S12: Gene structure of HD-Zip IV genes showing the exon-intron structure. (TIFF 633 KB)

Additional file 13: Table S1: List of homoeologous soybean HD-Zip genes. (XLSX 56 KB)

Additional file 14: Figure S13: Expression profiles of HD-Zip I genes in seven tissues of soybean. The Reads/Kb/Million (RPKM) normalized values of expressed genes was log_2_-transformed and visualized as heatmaps. Genes in the heatmap are ordered for consistency with the phylogeny in Figure 1. The abbreviation “SAM” in the tissue label represents “shoot apical meristem”. (TIFF 328 KB)

Additional file 15: Figure S14: Expression profiles of HD-Zip II genes in seven tissues of soybean. The Reads/Kb/Million (RPKM) normalized values of expressed genes was log_2_-transformed and visualized as heatmaps. Genes in the heatmap are ordered for consistency with the phylogeny in Figure 2. The abbreviation “SAM” in the tissue label represents “shoot apical meristem”. (TIFF 319 KB)

Additional file 16: Figure S15: Expression profiles of HD-Zip III genes in seven tissues of soybean. The Reads/Kb/Million (RPKM) normalized values of expressed genes was log_2_-transformed and visualized as heatmaps. Genes in the heatmap are ordered for consistency with the phylogeny in Figure 3. The abbreviation “SAM” in the tissue label represents “shoot apical meristem”. (TIFF 233 KB)

Additional file 17: Figure S16: Expression profiles of HD-Zip IV genes in seven tissues of soybean. The Reads/Kb/Million (RPKM) normalized values of expressed genes was log_2_-transformed and visualized as heatmaps. Genes in the heatmap are ordered for consistency with the phylogeny in Figure 4. The abbreviation “SAM” in the tissue label represents “shoot apical meristem”. (TIFF 326 KB)

Additional file 18: Figure S17: Expression profiles of HD-Zip I genes in mock-inoculated and *Bradyrhizobium japonicum*-infected root hair cells harvested at 12, 24, and 48 hr after inoculation (HAI), and stripped roots harvested at 48 HAI with *B. japonicum*. The Reads/Kb/Million (RPKM) normalized values of expressed genes was log_2_-transformed and visualized as heatmaps. Genes in the heatmap are ordered for consistency with the phylogeny in Figure 1. The abbreviation RH_UN and RH_IN in the tissue label represent mock-inoculated and *B. japonicum* infected root hair cells respectively. The sample RS_48HAI_IN represents stripped roots harvested at 48 HAI with *B. japonicum*. (TIFF 330 KB)

Additional file 19: Figure S18: Expression profiles of HD-Zip II genes in mock-inoculated and *Bradyrhizobium japonicum*-infected root hair cells harvested at 12, 24, and 48 hr after inoculation (HAI), and stripped roots harvested at 48 HAI with *B. japonicum*. The Reads/Kb/Million (RPKM) normalized values of expressed genes was log_2_-transformed and visualized as heatmaps. Genes in the heatmap are ordered for consistency with the phylogeny in Figure 2. The abbreviation RH_UN and RH_IN in the tissue label represent mock-inoculated and *B. japonicum* infected root hair cells respectively. The sample RS_48HAI_IN represents stripped roots harvested at 48 HAI with *B. japonicum*. (TIFF 319 KB)

Additional file 20: Figure S19: Expression profiles of HD-Zip III genes in mock-inoculated and *Bradyrhizobium japonicum*-infected root hair cells harvested at 12, 24, and 48 hr after inoculation (HAI), and stripped roots harvested at 48 HAI with *B. japonicum*. The Reads/Kb/Million (RPKM) normalized values of expressed genes was log_2_-transformed and visualized as heatmaps. Genes in the heatmap are ordered for consistency with the phylogeny in Figure 3. The abbreviation RH_UN and RH_IN in the tissue label represent mock-inoculated and *B. japonicum* infected root hair cells respectively. The sample RS_48HAI_IN represents stripped roots harvested at 48 HAI with *B. japonicum*. (TIFF 240 KB)

Additional file 21: Figure S20: Expression profiles of HD-Zip IV genes in mock-inoculated and *Bradyrhizobium japonicum*-infected root hair cells harvested at 12, 24, and 48 hr after inoculation (HAI), and stripped roots harvested at 48 HAI with *B. japonicum*. The Reads/Kb/Million (RPKM) normalized values of expressed genes was log_2_-transformed and visualized as heatmaps. Genes in the heatmap are ordered for consistency with the phylogeny in Figure 4. The abbreviation RH_UN and RH_IN in the tissue label represent mock-inoculated and *B. japonicum* infected root hair cells respectively. The sample RS_48HAI_IN represents stripped roots harvested at 48 HAI with *B. japonicum*. (TIFF 326 KB)

Additional file 22: Table S2: Soybean genes differentially expressed under dehydration stress at 1 hr. The table includes mean expression values under control and stress conditions; fold change and log_2_ fold change values, *P*-values and adjusted *P*-values, and residual variance quotients of control and treatment samples. See Methods for the criteria of differential expression. (TXT 396 KB)

Additional file 23: Table S3: Soybean genes differentially expressed under dehydration stress at 6 hr. The table includes mean expression values under control and stress conditions; fold change and log_2_ fold change values, *P*-values and adjusted *P*-values, and residual variance quotients of control and treatment samples. See Methods for the criteria of differential expression. (TXT 395 KB)

Additional file 24: Table S4: Soybean genes differentially expressed under dehydration stress at 12 hr. The table includes mean expression values under control and stress conditions; fold change and log_2_ fold change values, *P*-values and adjusted *P*-values, and residual variance quotients of control and treatment samples. See Methods for the criteria of differential expression. (TXT 326 KB)

Additional file 25: Table S5: Soybean genes differentially expressed under salt stress at 1 hr. The table includes mean expression values under control and stress conditions; fold change and log_2_ fold change values, *P*-values and adjusted *P*-values, and residual variance quotients of control and treatment samples. See Methods for the criteria of differential expression. (TXT 291 KB)

Additional file 26: Table S6: Soybean genes differentially expressed under salt stress at 6 hr. The table includes mean expression values under control and stress conditions; fold change and log_2_ fold change values, *P*-values and adjusted *P*-values, and residual variance quotients of control and treatment samples. See Methods for the criteria of differential expression. (TXT 741 KB)

Additional file 27: Table S7: Soybean genes differentially expressed under salt stress at 12 hr. The table includes mean expression values under control and stress conditions; fold change and log_2_ fold change values, *P*-values and adjusted *P*-values, and residual variance quotients of control and treatment samples. See Methods for the criteria of differential expression. (TXT 1 MB)

Additional file 28: Table S8: Summary statistics of RNA-Seq analysis under dehydration and salt stress. (XLSX 39 KB)

Additional file 29: Table S9: Raw read counts for each of the soybean gene under dehydration and salt stress at 0, 1, 6 and 12 hr generated in the RNA-Seq experiment. (TXT 4 MB)

Additional file 30: Table S10: DESeq normalized read counts for each of the soybean gene under dehydration and salt stress at 0, 1, 6 and 12 hr generated in the RNA-Seq experiment. (TXT 16 MB)

Additional file 31: Table S11: List of GO biological process and molecular function terms significantly (corrected *P* <0.05) overrepresented in differentially expressed genes under dehydration and salt stress. (XLSX 55 KB)

Additional file 32: Table S12: List of transcription factor classes significantly (corrected *P* <0.05) overrepresented in differentially expressed genes under dehydration and salt stress. (TXT 32 KB)

Additional file 33: Table S13: List of plant transcription factor binding sites (TFBSs) significantly (*P* <0.05, motif score >5) overrepresented in the promoters of HD-Zip genes, differentially expressed (DE) genes under dehydration and salt stress, and their respective counts. The TFBSs are provided separately for each of the HD-Zip gene and DE genes. (TXT 4 MB)

Additional file 34: Table S14: List of plant transcription factor binding sites (TFBSs) significantly (*P* <0.05, motif score >5) overrepresented in the promoters of differentially expressed genes under dehydration and salt stress, with relative proportion of each TFBS under each of the stress treatment. The list of genes that were excluded from the analysis because they did not meet the selection criteria (see Methods for selection criteria) are included. (XLSX 48 KB)
