# Supplementary figures and images for: Comprehensive characterization and RNA-Seq profiling of the HD-Zip transcription factor family in soybean (Glycine max) during dehydration and salt stress
Source: BMC Genomics. 2014 Nov 3;15:950. doi: 10.1186/1471-2164-15-950 (PMC4226900; doi:10.1186/1471-2164-15-950)

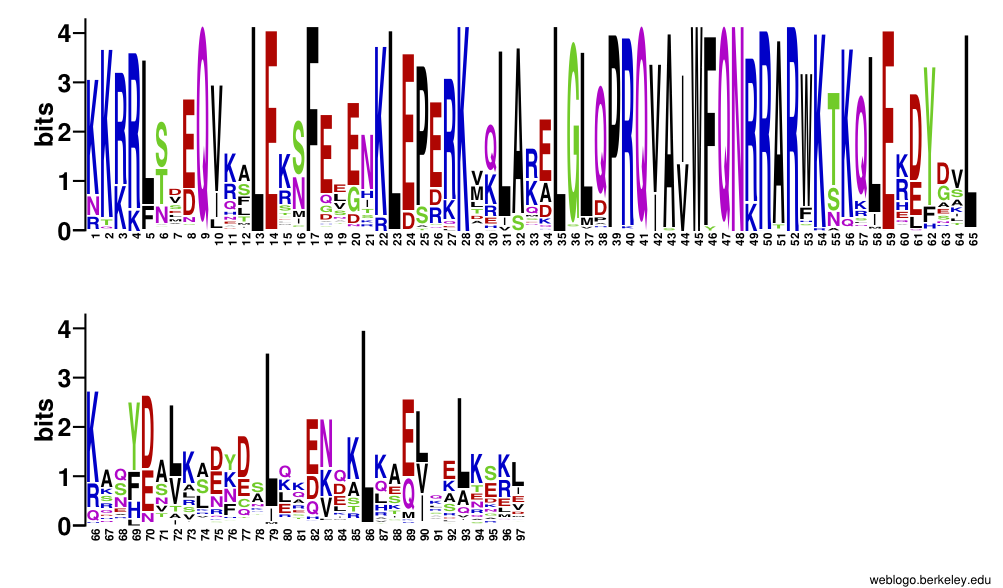

Supplement: Supplementary file 1 — Additional file 1: Figure S1: Sequence logo of HD-Zip I displaying the conserved residues in HMM alignment. (TIFF 246 KB) [file 12864_2014_6641_MOESM1_ESM.tiff]

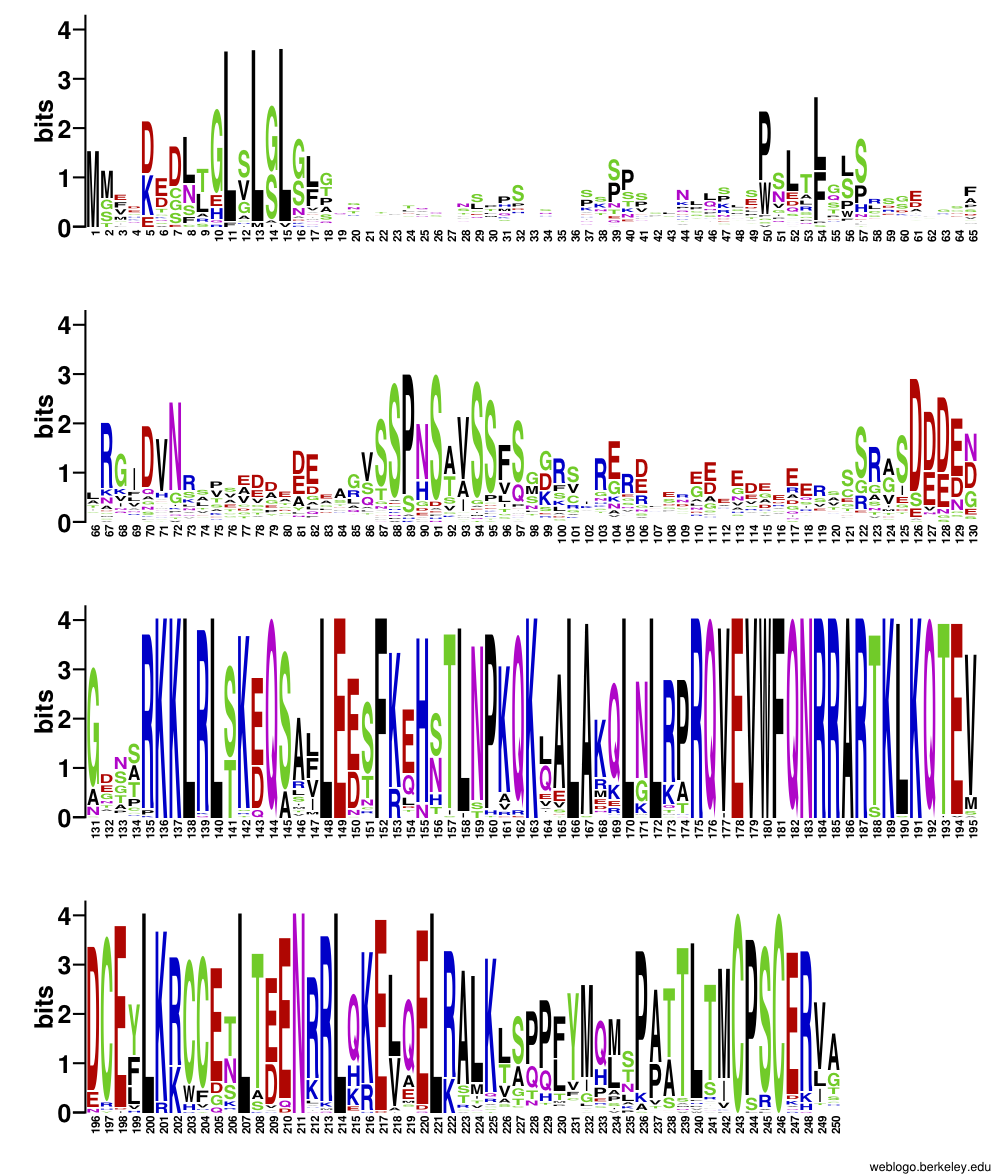

Supplement: Supplementary file 2 — Additional file 2: Figure S2: Sequence logo of HD-Zip II displaying the conserved residues in HMM alignment. (TIFF 525 KB) [file 12864_2014_6641_MOESM2_ESM.tiff]

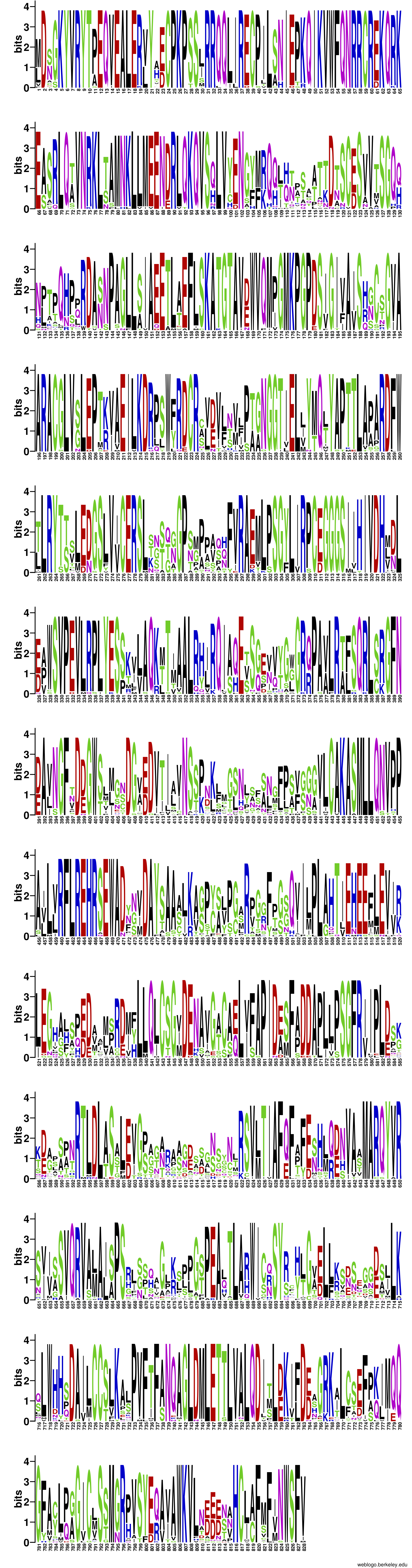

Supplement: Supplementary file 3 — Additional file 3: Figure S3: Sequence logo of HD-Zip III displaying the conserved residues in HMM alignment. (TIFF 5 MB) [file 12864_2014_6641_MOESM3_ESM.tiff]

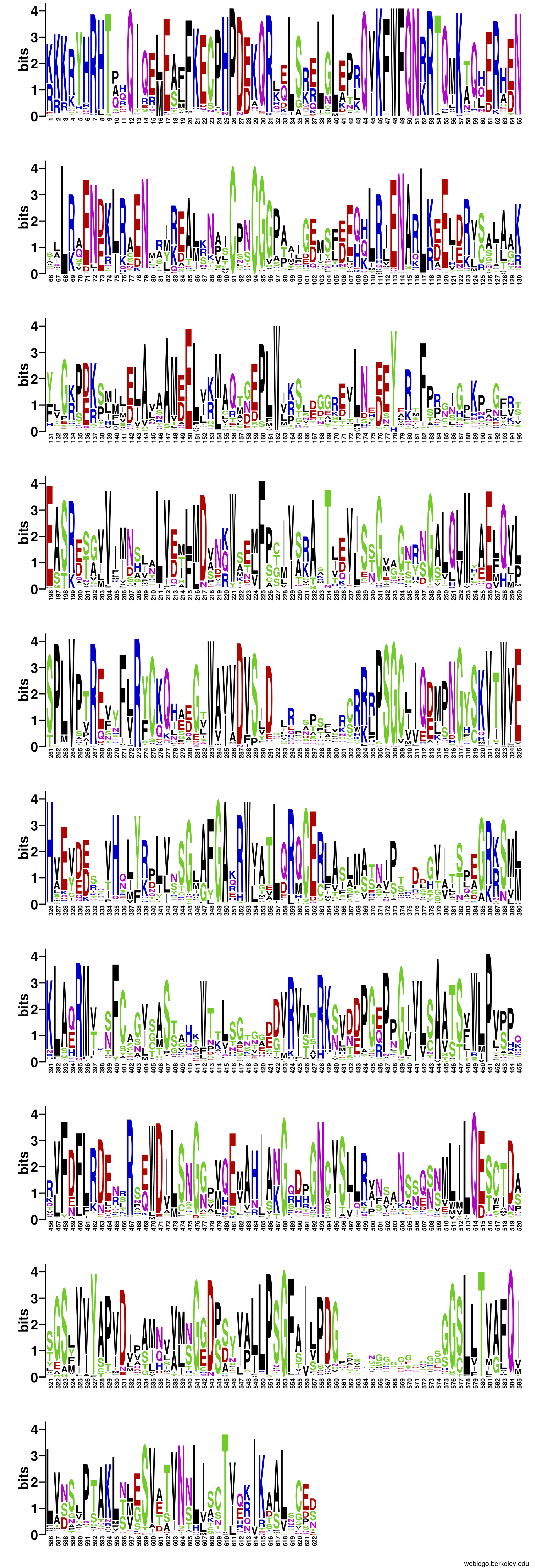

Supplement: Supplementary file 4 — Additional file 4: Figure S4: Sequence logo of HD-Zip IV displaying the conserved residues in HMM alignment. (TIFF 3 MB) [file 12864_2014_6641_MOESM4_ESM.tiff]

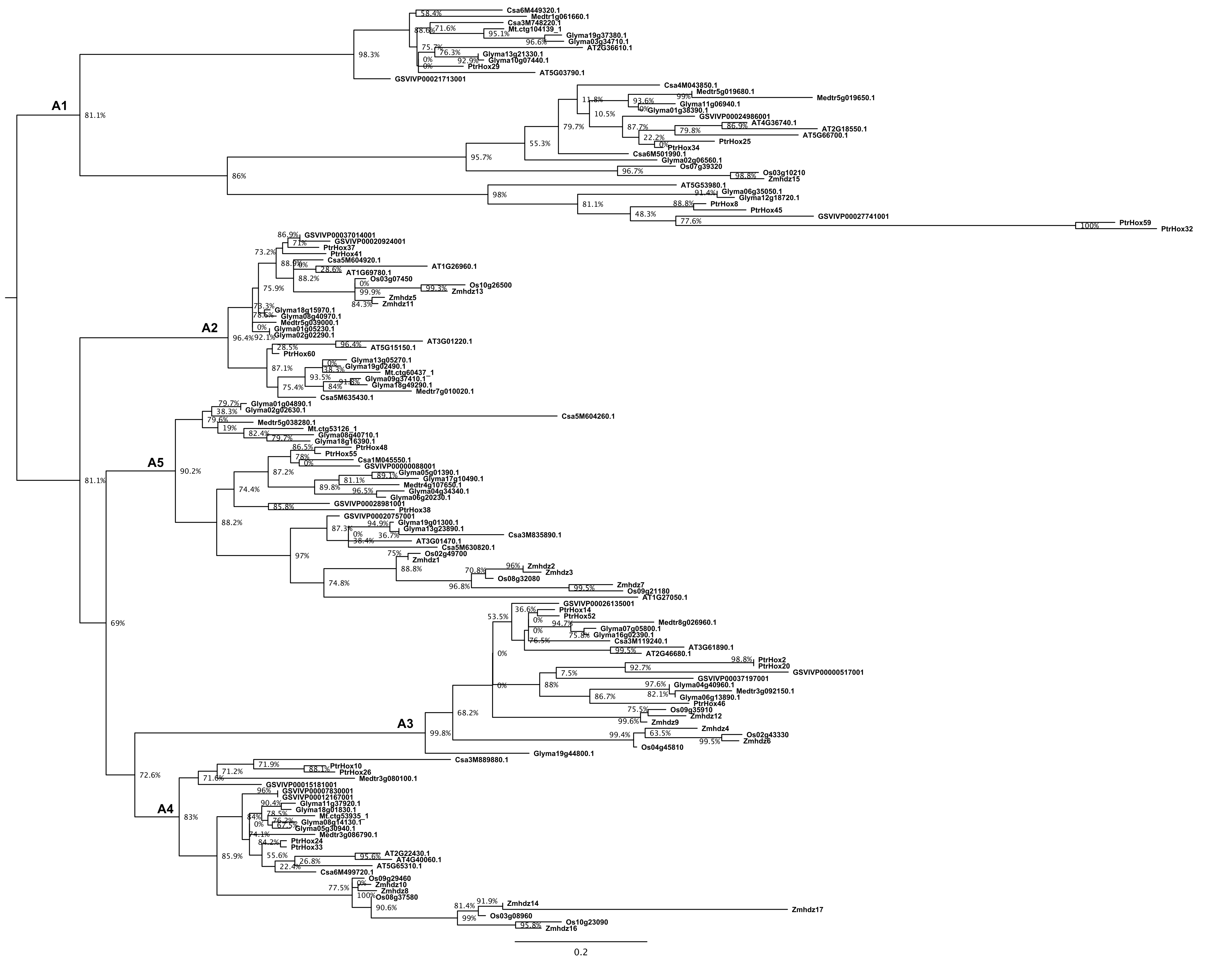

Supplement: Supplementary file 5 — Additional file 5: Figure S5: Phylogenetic relationships of HD-Zip I proteins from soybean, Medicago, Arabidopsis, grape, poplar, cucumber, maize and rice. The phylogenetic tree was built using the maximum likelihood method implemented in PhyML. The letters A1- A5 represent ancient angiosperm clades, based on whole genome duplication events, and the copy number of genes from each of the species. The letters are ordered for consistency with the phylogeny in Figure 1. The branch support values estimated using approximate likelihood ratio test (aLRT) are displayed in percentages. Rooting of the tree was inferred from Ariel et al. [1], angiosperm clade composition, and outgroup sequences from other subfamilies. (TIFF 1 MB) [file 12864_2014_6641_MOESM5_ESM.tiff]

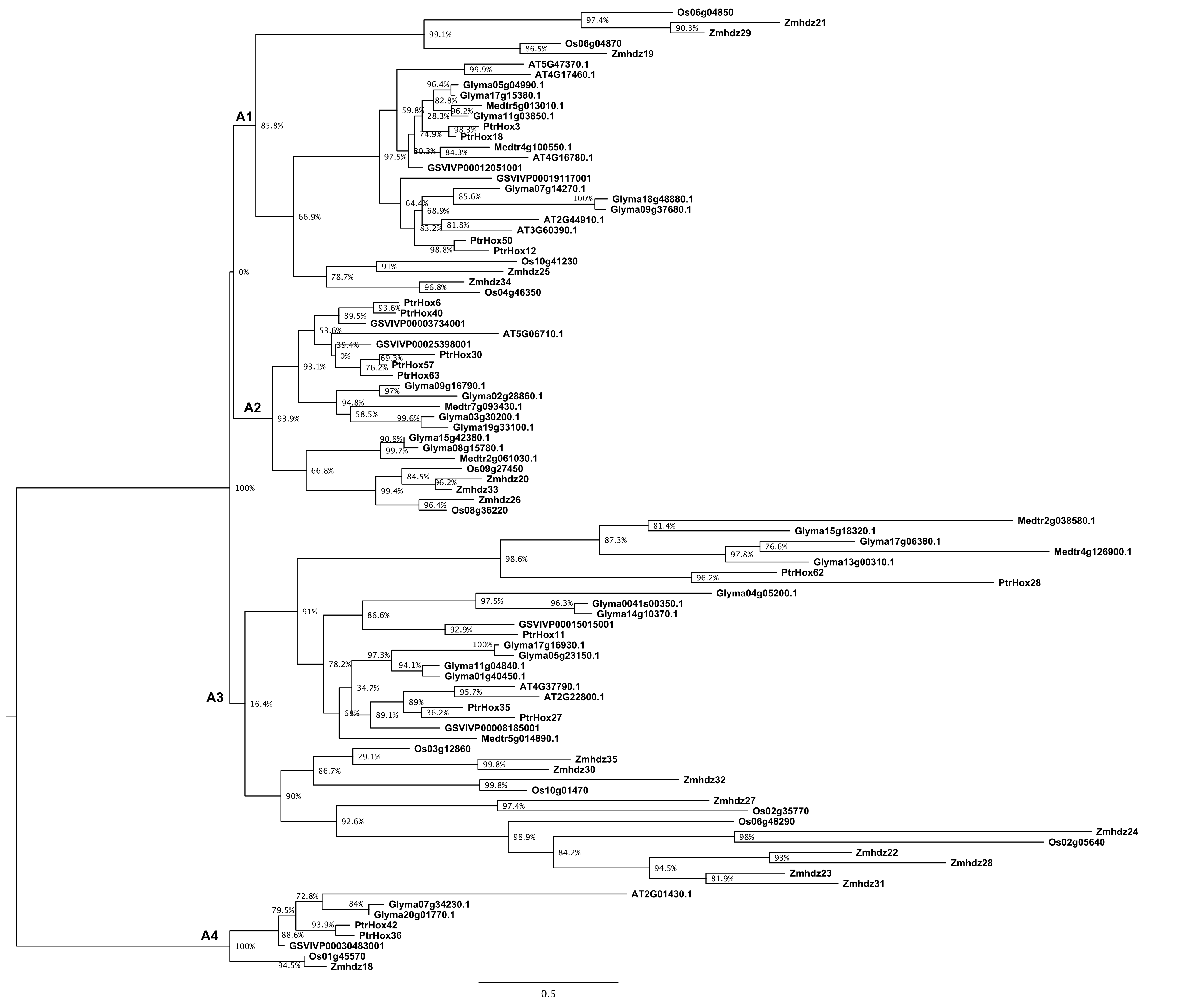

Supplement: Supplementary file 6 — Additional file 6: Figure S6: Phylogenetic relationships of HD-Zip II proteins from soybean, Medicago, Arabidopsis, grape, poplar, maize and rice. The phylogenetic tree was built using the maximum likelihood method implemented in PhyML. The letters A1- A4 represent ancient angiosperm clades, based on whole genome duplication events, and the copy number of genes from each of the species. The letters are ordered for consistency with the phylogeny in Figure 2. The branch support values estimated using approximate likelihood ratio test (aLRT) are displayed in percentages. Rooting of the tree was inferred from Ariel et al. [1], angiosperm clade composition, and outgroup sequences from other subfamilies. (TIFF 994 KB) [file 12864_2014_6641_MOESM6_ESM.tiff]

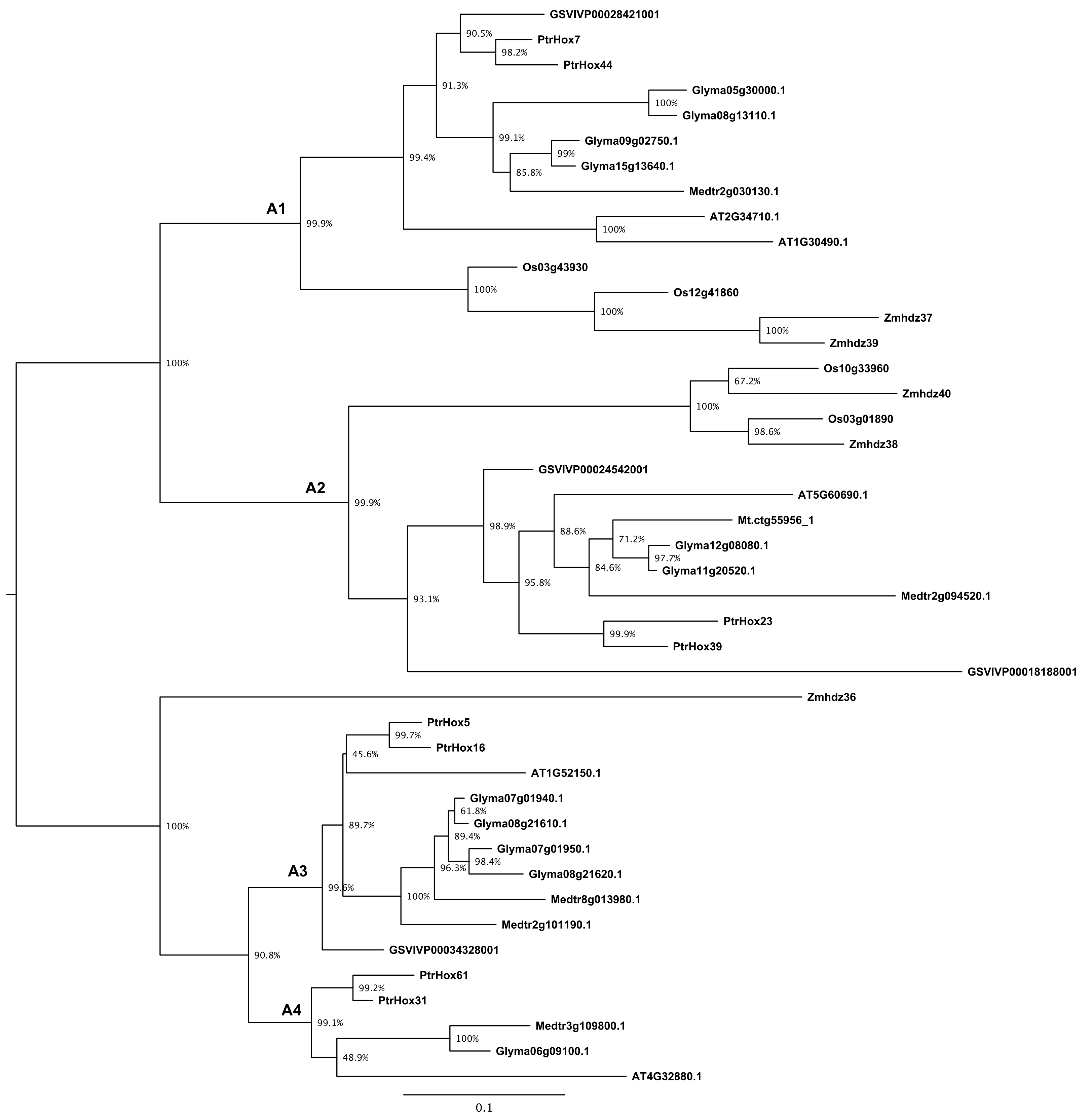

Supplement: Supplementary file 7 — Additional file 7: Figure S7: Phylogenetic relationships of HD-Zip III proteins from soybean, Medicago, Arabidopsis, grape, poplar, maize and rice. The phylogenetic tree was built using the maximum likelihood method implemented in PhyML. The letters A1- A4 represent ancient angiosperm clades, based on whole genome duplication events, and the copy number of genes from each of the species. The letters are ordered for consistency with the phylogeny in Figure 3. The branch support values estimated using approximate likelihood ratio test (aLRT) are displayed in percentages. Rooting of the tree was inferred from Ariel et al. [1], angiosperm clade composition, and outgroup sequences from other subfamilies. (TIFF 549 KB) [file 12864_2014_6641_MOESM7_ESM.tiff]

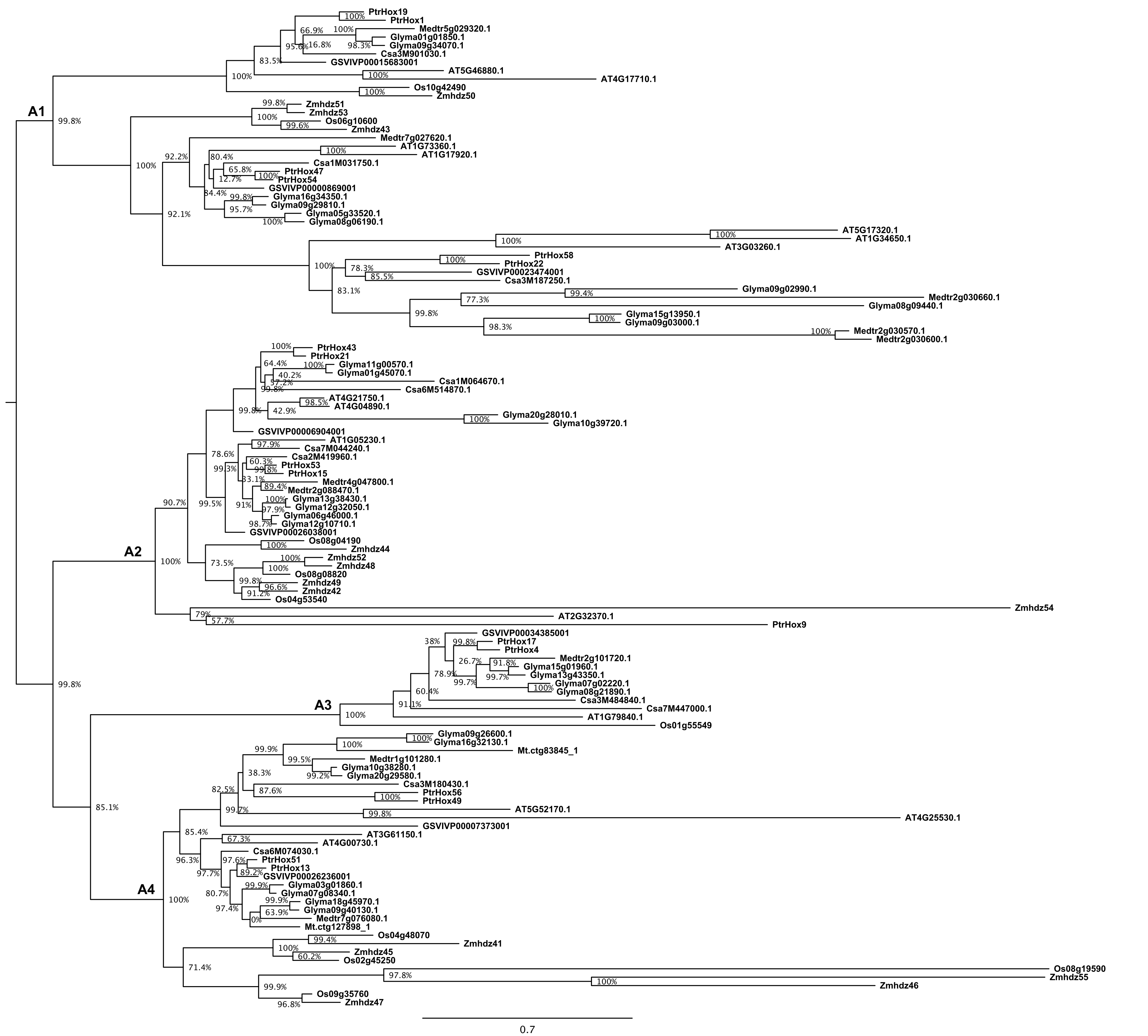

Supplement: Supplementary file 8 — Additional file 8: Figure S8: Phylogenetic relationships of HD-Zip IV proteins from soybean, Medicago, Arabidopsis, grape, poplar, cucumber, maize and rice. The phylogenetic tree was built using the maximum likelihood method implemented in PhyML. The letters A1- A4 represent ancient angiosperm clades, based on whole genome duplication events, and the copy number of genes from each of the species. The letters are ordered for consistency with the phylogeny in Figure 4. The branch support values estimated using approximate likelihood ratio test (aLRT) are displayed in percentages. Rooting of the tree was inferred from Ariel et al. [1], angiosperm clade composition, and outgroup sequences from other subfamilies. Genes Medtr5g005600.1 and Os01g57890 belong to the angiosperm clade “A2”. These two genes are not shown in the phylogeny because adding them significantly affects the topology. (TIFF 1 MB) [file 12864_2014_6641_MOESM8_ESM.tiff]

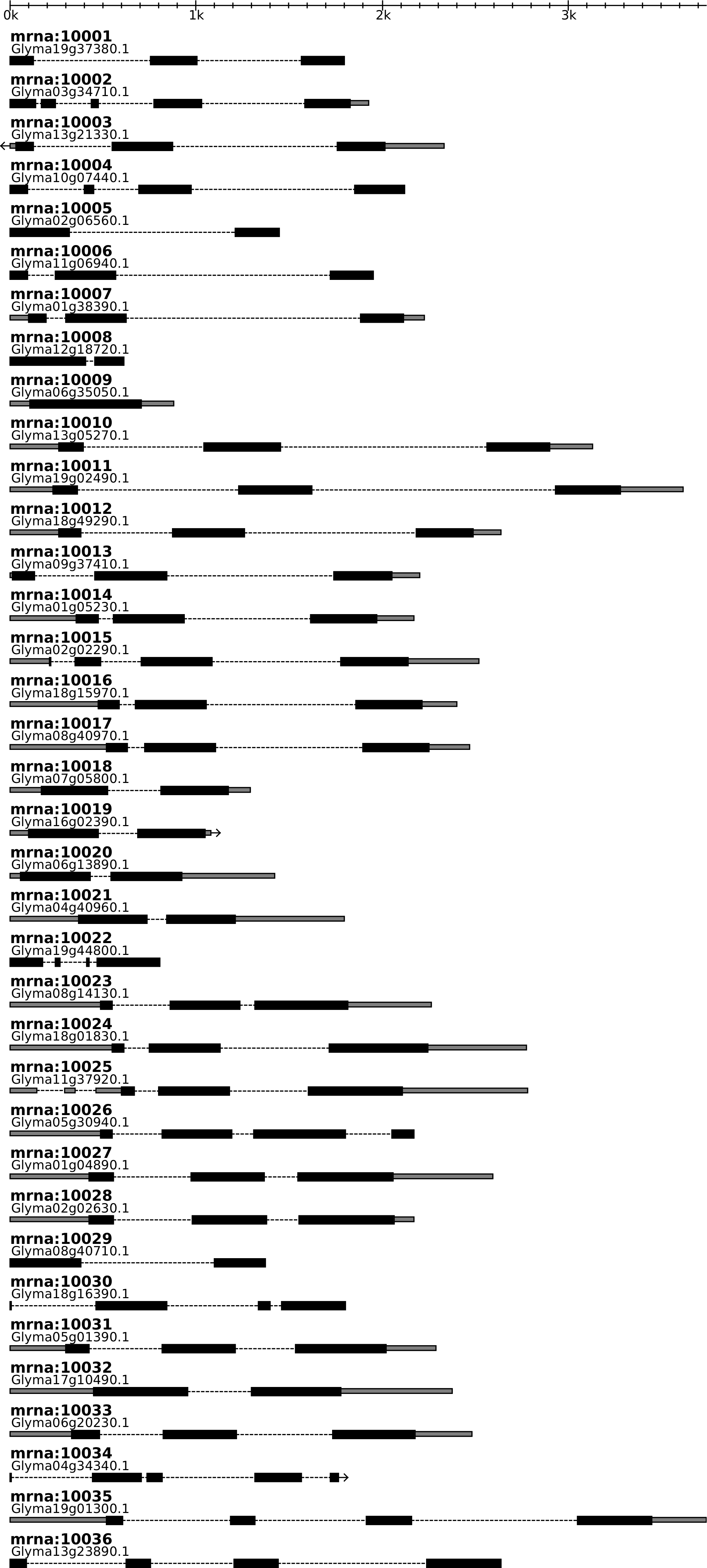

Supplement: Supplementary file 9 — Additional file 9: Figure S9: Gene structure of HD-Zip I genes showing the exon-intron structure. (TIFF 676 KB) [file 12864_2014_6641_MOESM9_ESM.tiff]

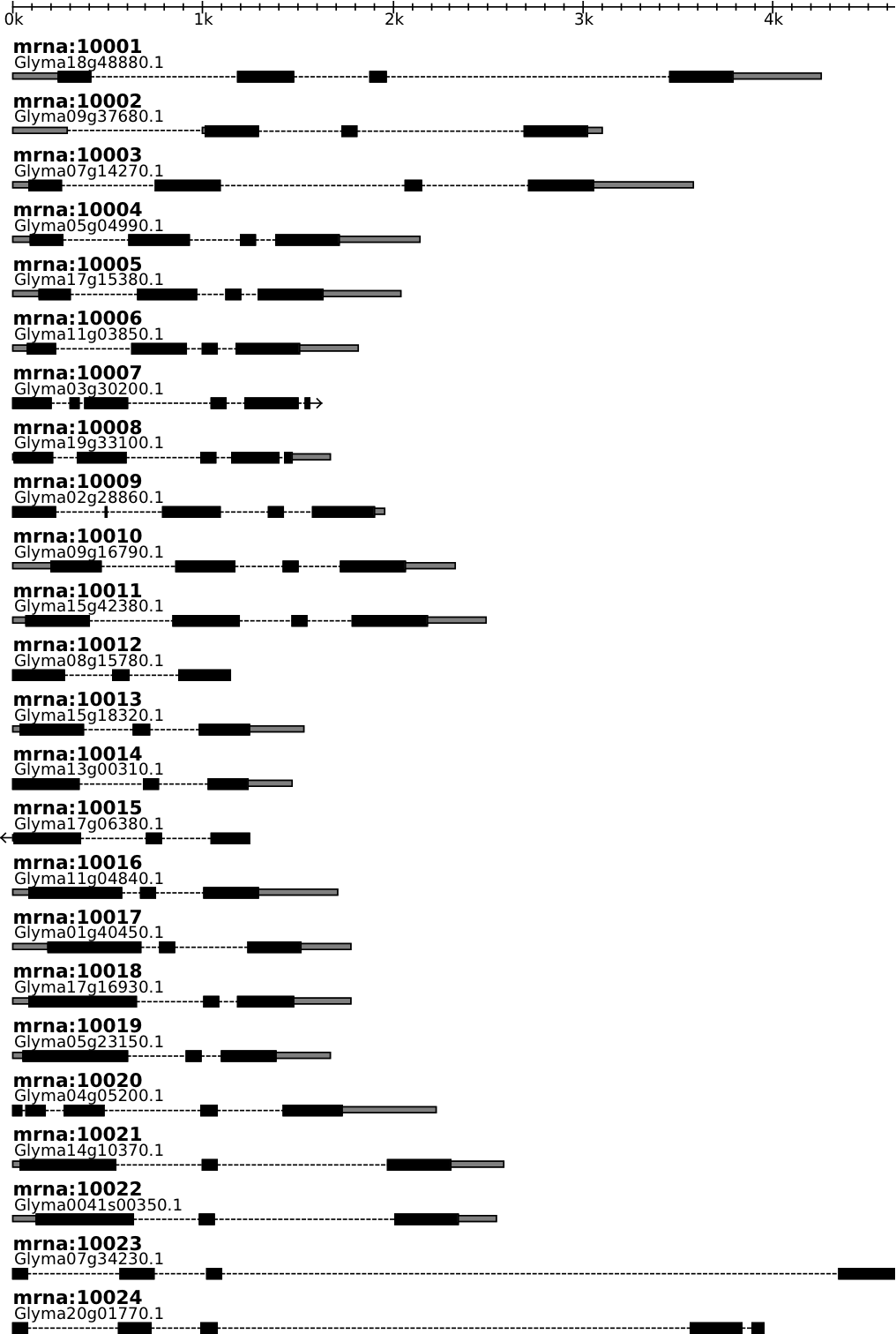

Supplement: Supplementary file 10 — Additional file 10: Figure S10: Gene structure of HD-Zip II genes showing the exon-intron structure. (TIFF 193 KB) [file 12864_2014_6641_MOESM10_ESM.tiff]

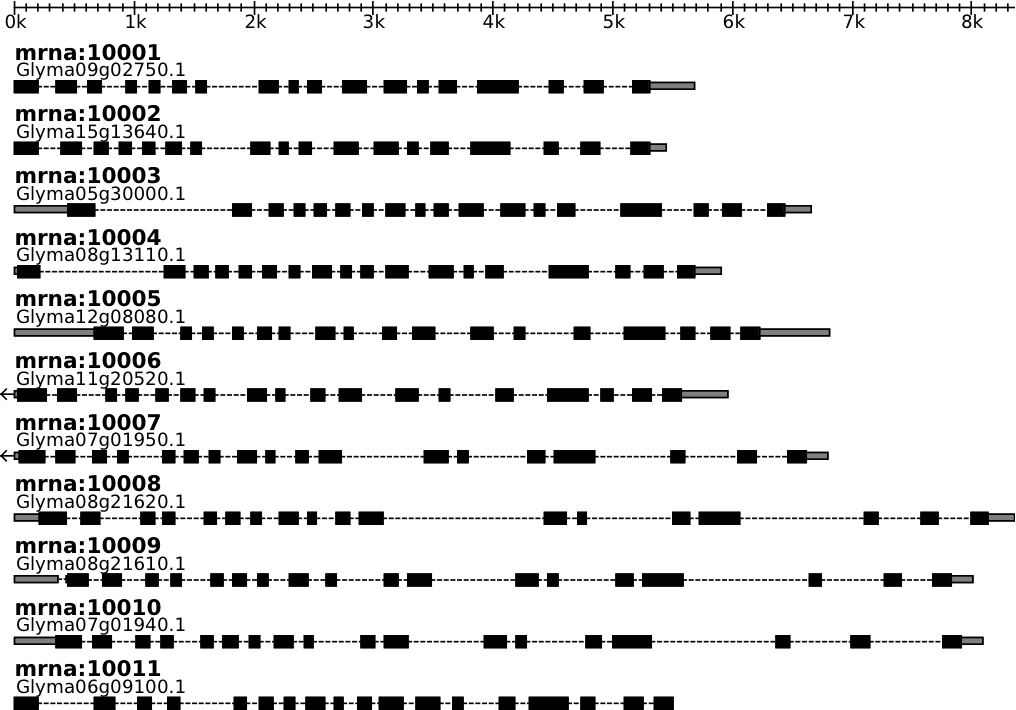

Supplement: Supplementary file 11 — Additional file 11: Figure S11: Gene structure of HD-Zip III genes showing the exon-intron structure. (TIFF 114 KB) [file 12864_2014_6641_MOESM11_ESM.tiff]

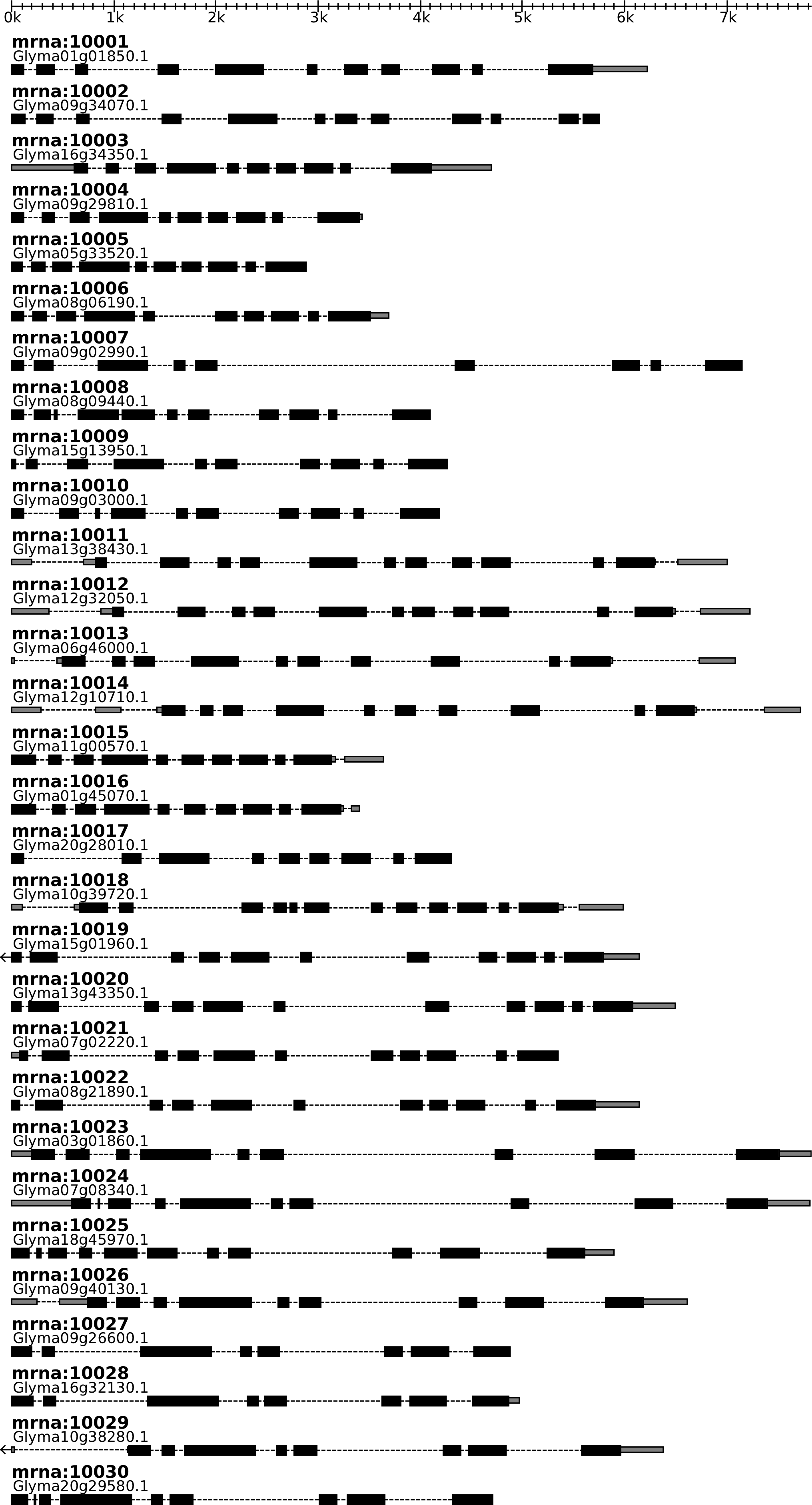

Supplement: Supplementary file 12 — Additional file 12: Figure S12: Gene structure of HD-Zip IV genes showing the exon-intron structure. (TIFF 633 KB) [file 12864_2014_6641_MOESM12_ESM.tiff]

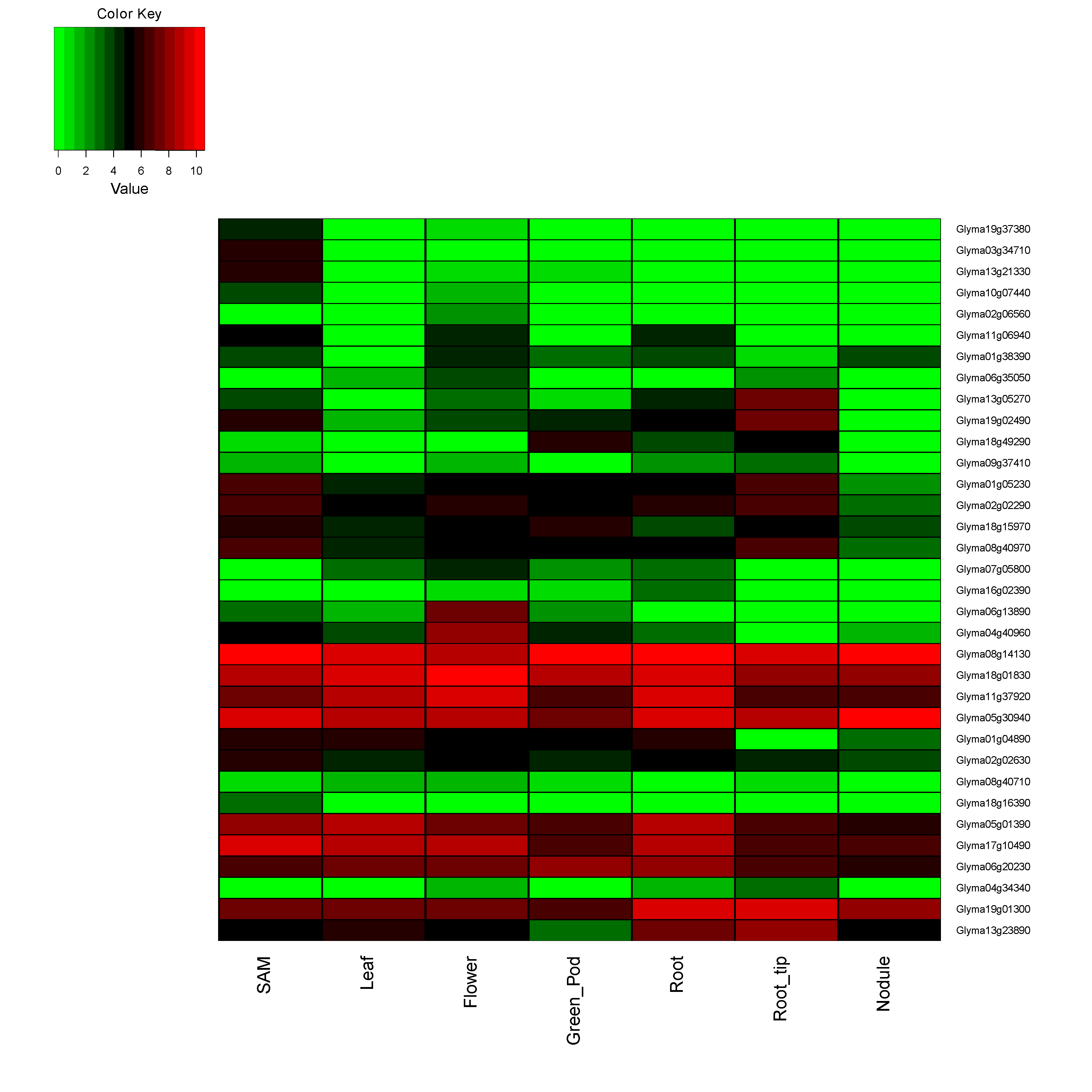

Supplement: Supplementary file 14 — Additional file 14: Figure S13: Expression profiles of HD-Zip I genes in seven tissues of soybean. The Reads/Kb/Million (RPKM) normalized values of expressed genes was log2-transformed and visualized as heatmaps. Genes in the heatmap are ordered for consistency with the phylogeny in Figure 1. The abbreviation “SAM” in the tissue label represents “shoot apical meristem”. (TIFF 328 KB) [file 12864_2014_6641_MOESM14_ESM.tiff]

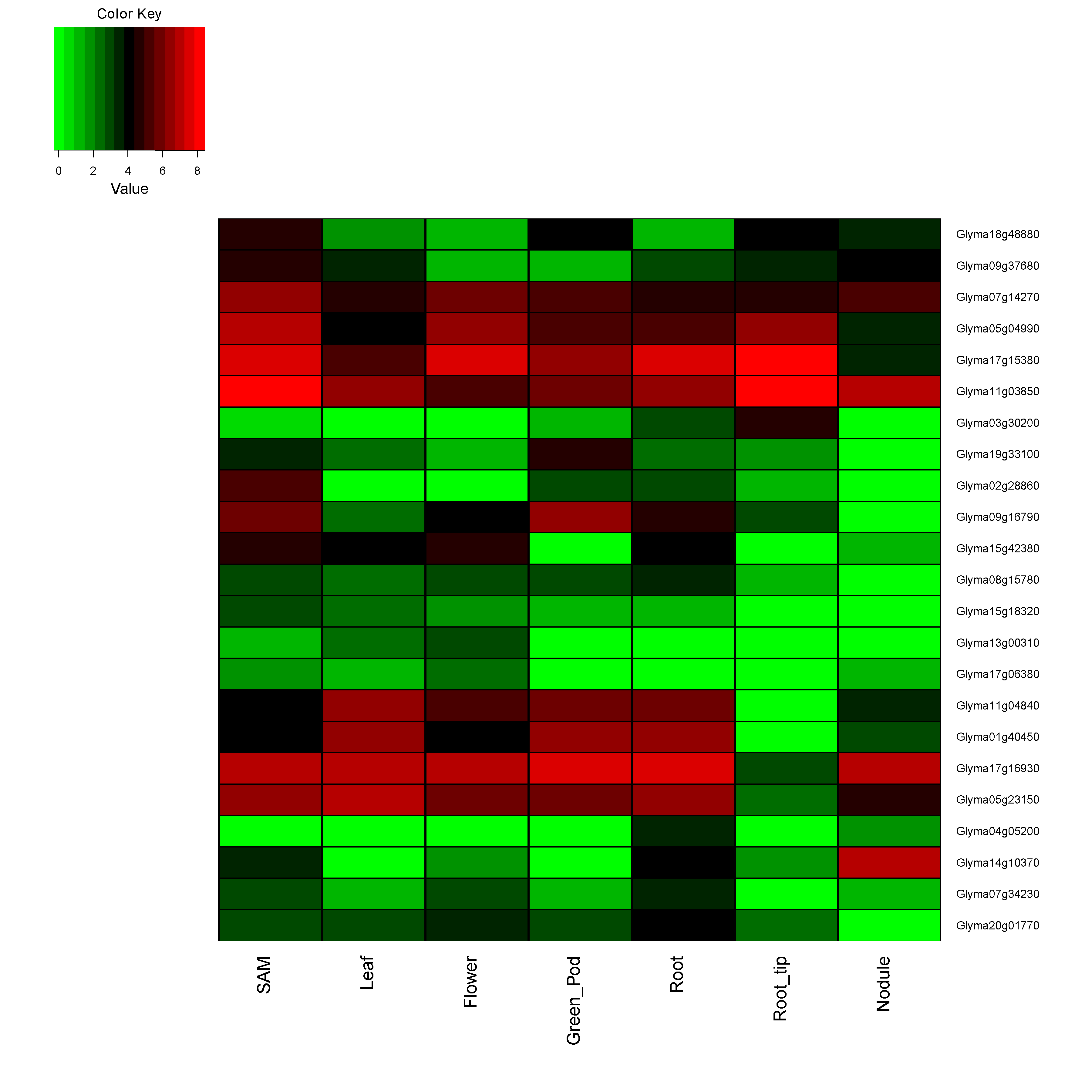

Supplement: Supplementary file 15 — Additional file 15: Figure S14: Expression profiles of HD-Zip II genes in seven tissues of soybean. The Reads/Kb/Million (RPKM) normalized values of expressed genes was log2-transformed and visualized as heatmaps. Genes in the heatmap are ordered for consistency with the phylogeny in Figure 2. The abbreviation “SAM” in the tissue label represents “shoot apical meristem”. (TIFF 319 KB) [file 12864_2014_6641_MOESM15_ESM.tiff]

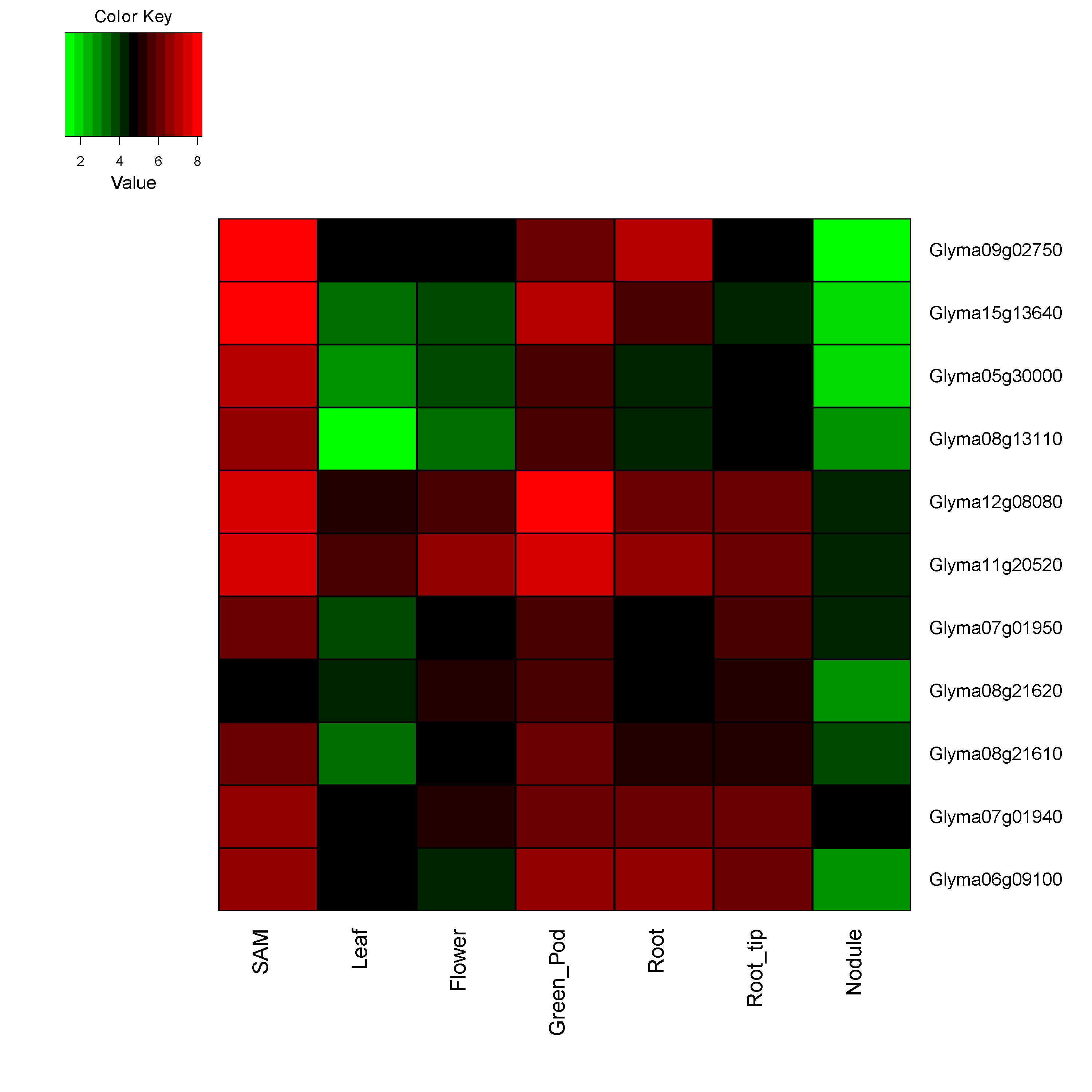

Supplement: Supplementary file 16 — Additional file 16: Figure S15: Expression profiles of HD-Zip III genes in seven tissues of soybean. The Reads/Kb/Million (RPKM) normalized values of expressed genes was log2-transformed and visualized as heatmaps. Genes in the heatmap are ordered for consistency with the phylogeny in Figure 3. The abbreviation “SAM” in the tissue label represents “shoot apical meristem”. (TIFF 233 KB) [file 12864_2014_6641_MOESM16_ESM.tiff]

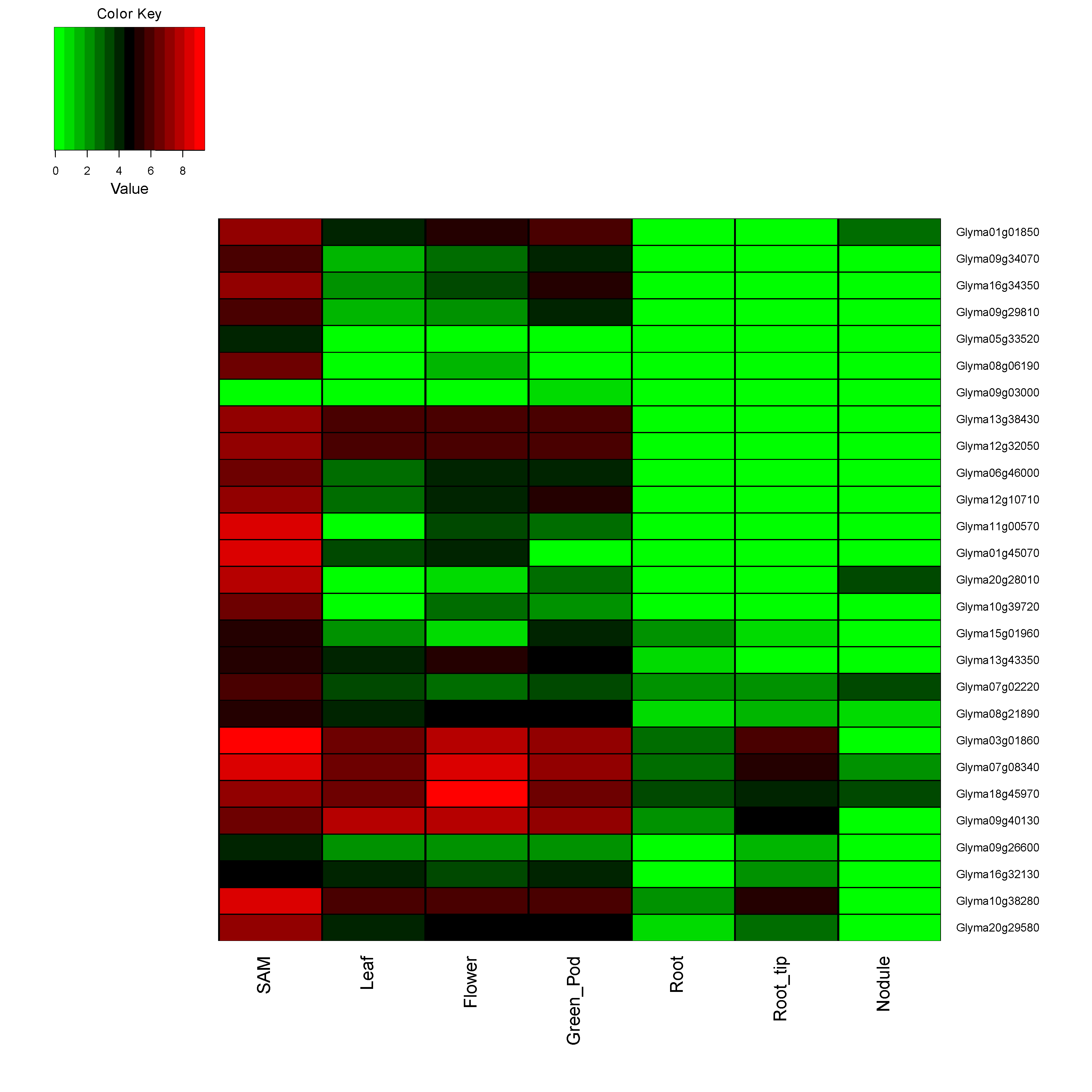

Supplement: Supplementary file 17 — Additional file 17: Figure S16: Expression profiles of HD-Zip IV genes in seven tissues of soybean. The Reads/Kb/Million (RPKM) normalized values of expressed genes was log2-transformed and visualized as heatmaps. Genes in the heatmap are ordered for consistency with the phylogeny in Figure 4. The abbreviation “SAM” in the tissue label represents “shoot apical meristem”. (TIFF 326 KB) [file 12864_2014_6641_MOESM17_ESM.tiff]

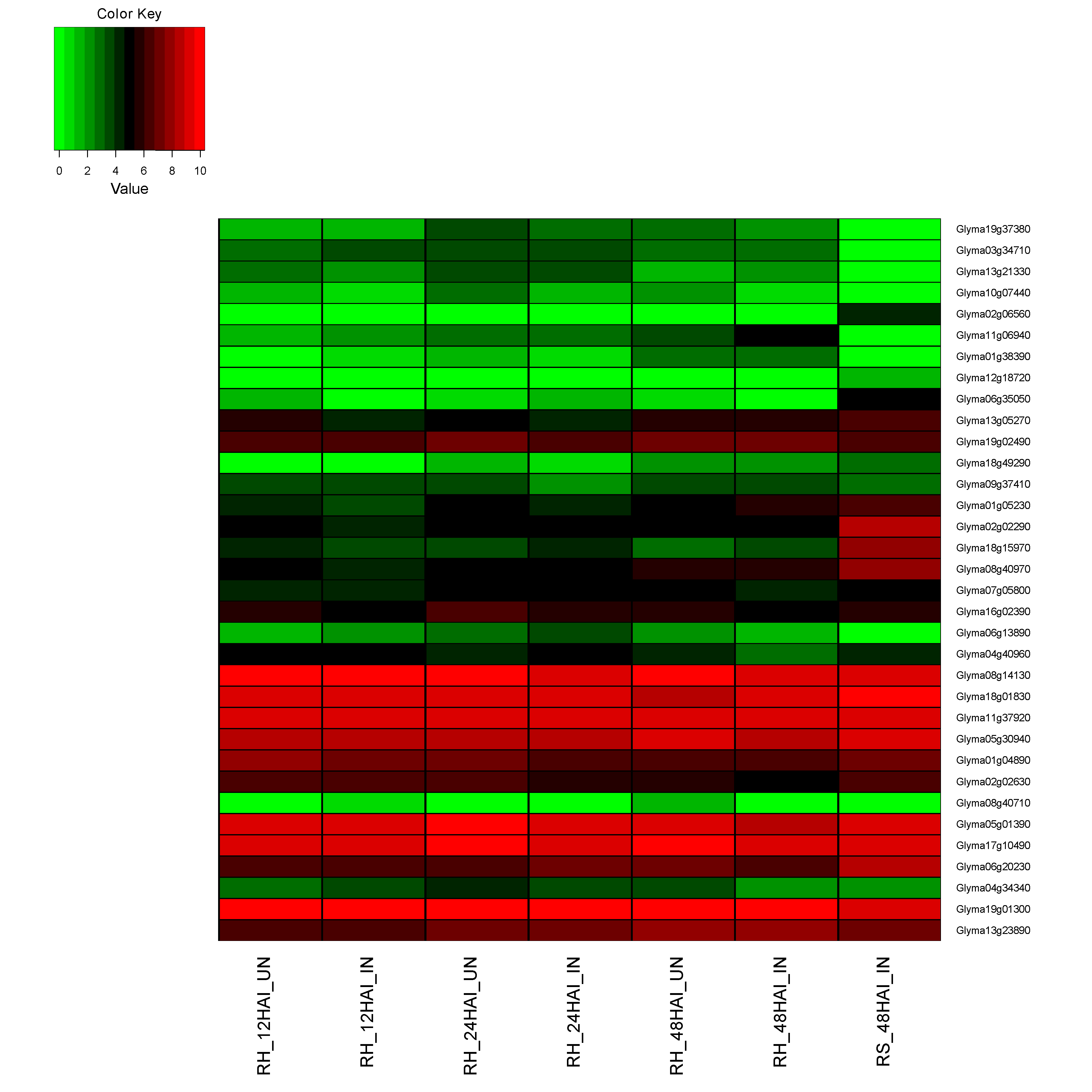

Supplement: Supplementary file 18 — Additional file 18: Figure S17: Expression profiles of HD-Zip I genes in mock-inoculated and Bradyrhizobium japonicum-infected root hair cells harvested at 12, 24, and 48 hr after inoculation (HAI), and stripped roots harvested at 48 HAI with B. japonicum. The Reads/Kb/Million (RPKM) normalized values of expressed genes was log2-transformed and visualized as heatmaps. Genes in the heatmap are ordered for consistency with the phylogeny in Figure 1. The abbreviation RH_UN and RH_IN in the tissue label represent mock-inoculated and B. japonicum infected root hair cells respectively. The sample RS_48HAI_IN represents stripped roots harvested at 48 HAI with B. japonicum. (TIFF 330 KB) [file 12864_2014_6641_MOESM18_ESM.tiff]

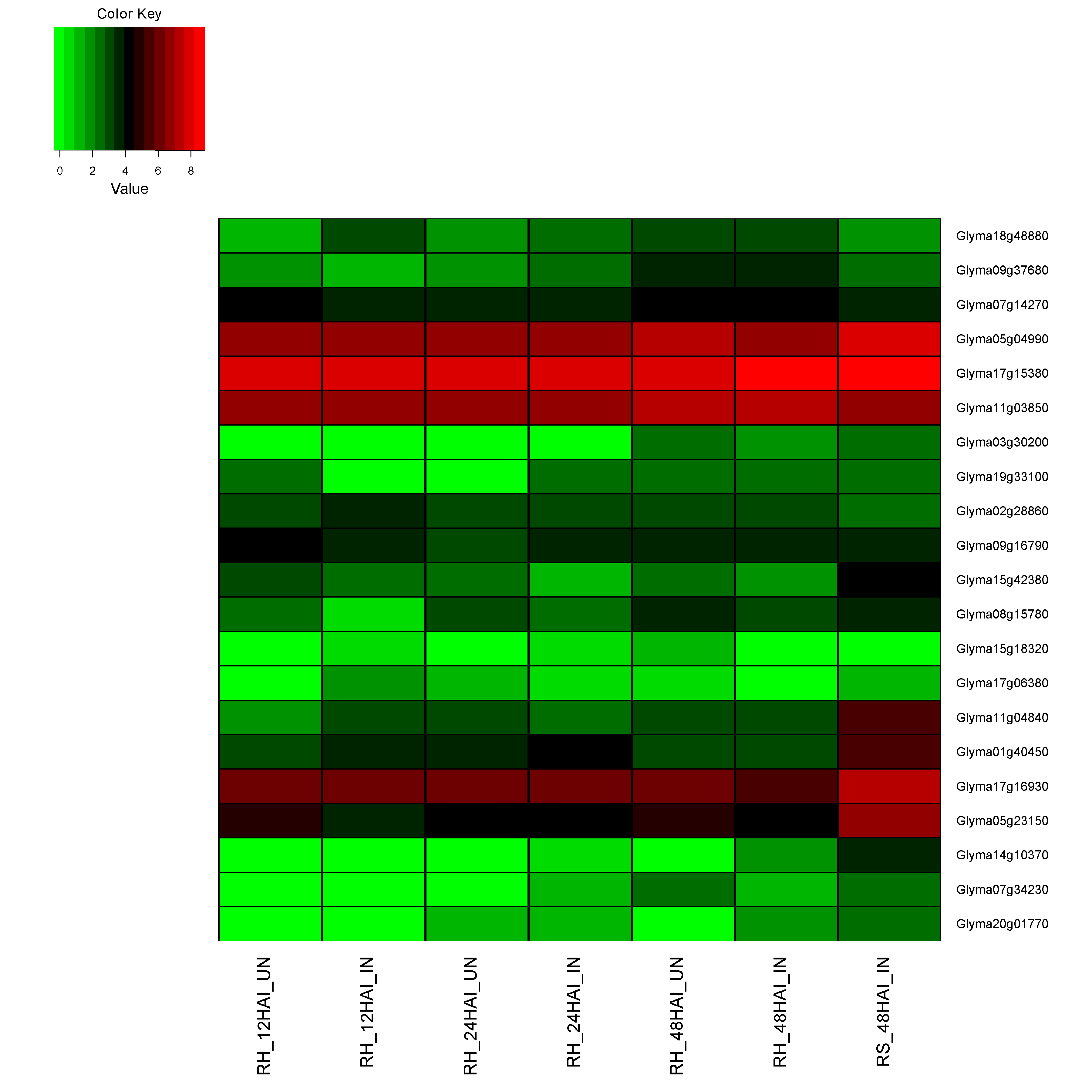

Supplement: Supplementary file 19 — Additional file 19: Figure S18: Expression profiles of HD-Zip II genes in mock-inoculated and Bradyrhizobium japonicum-infected root hair cells harvested at 12, 24, and 48 hr after inoculation (HAI), and stripped roots harvested at 48 HAI with B. japonicum. The Reads/Kb/Million (RPKM) normalized values of expressed genes was log2-transformed and visualized as heatmaps. Genes in the heatmap are ordered for consistency with the phylogeny in Figure 2. The abbreviation RH_UN and RH_IN in the tissue label represent mock-inoculated and B. japonicum infected root hair cells respectively. The sample RS_48HAI_IN represents stripped roots harvested at 48 HAI with B. japonicum. (TIFF 319 KB) [file 12864_2014_6641_MOESM19_ESM.tiff]

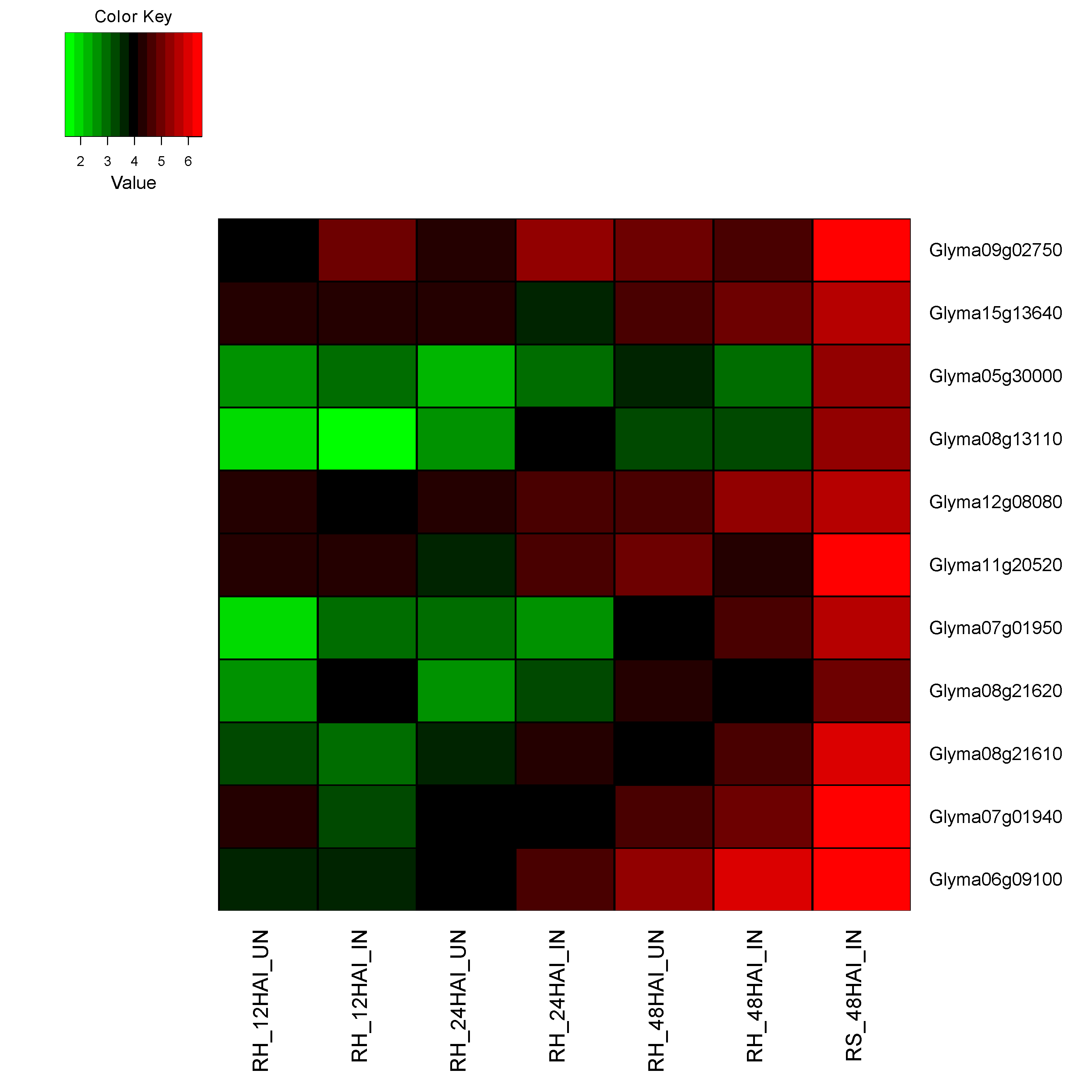

Supplement: Supplementary file 20 — Additional file 20: Figure S19: Expression profiles of HD-Zip III genes in mock-inoculated and Bradyrhizobium japonicum-infected root hair cells harvested at 12, 24, and 48 hr after inoculation (HAI), and stripped roots harvested at 48 HAI with B. japonicum. The Reads/Kb/Million (RPKM) normalized values of expressed genes was log2-transformed and visualized as heatmaps. Genes in the heatmap are ordered for consistency with the phylogeny in Figure 3. The abbreviation RH_UN and RH_IN in the tissue label represent mock-inoculated and B. japonicum infected root hair cells respectively. The sample RS_48HAI_IN represents stripped roots harvested at 48 HAI with B. japonicum. (TIFF 240 KB) [file 12864_2014_6641_MOESM20_ESM.tiff]

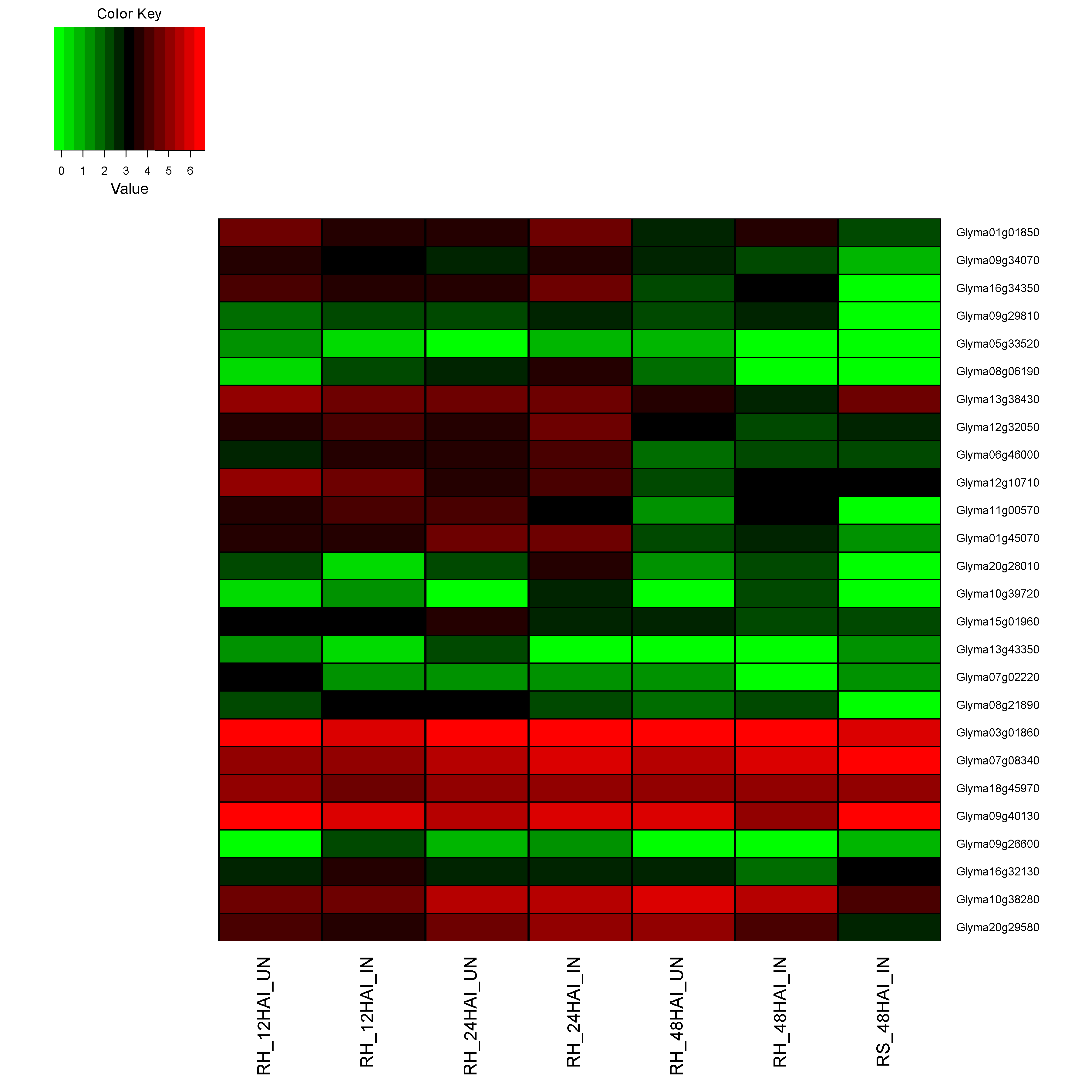

Supplement: Supplementary file 21 — Additional file 21: Figure S20: Expression profiles of HD-Zip IV genes in mock-inoculated and Bradyrhizobium japonicum-infected root hair cells harvested at 12, 24, and 48 hr after inoculation (HAI), and stripped roots harvested at 48 HAI with B. japonicum. The Reads/Kb/Million (RPKM) normalized values of expressed genes was log2-transformed and visualized as heatmaps. Genes in the heatmap are ordered for consistency with the phylogeny in Figure 4. The abbreviation RH_UN and RH_IN in the tissue label represent mock-inoculated and B. japonicum infected root hair cells respectively. The sample RS_48HAI_IN represents stripped roots harvested at 48 HAI with B. japonicum. (TIFF 326 KB) [file 12864_2014_6641_MOESM21_ESM.tiff]
